# Machine learning to predict plasma-based CO_2_ conversion in dielectric barrier discharge reactors

**DOI:** 10.1039/d6gc01077f

**Published:** 2026-05-14

**Authors:** Jiayin Li, Xinpei Lu, Pranav Arun, Jing Xu, Fausto Gallucci, Sirui Li, Annemie Bogaerts

**Affiliations:** a Research Group PLASMANT and Center of Excellence PLASMA, University of Antwerp, Department of Chemistry Antwerp 2610 Belgium annemie.bogaerts@uantwerpen.be; b Electrification Institute, University of Antwerp Olieweg 97 2020 Antwerp Belgium; c School of Electrical and Electronic Engineering, Huazhong University of Science and Technology Wuhan Hubei 430074 China; d Department of Chemical Engineering and Chemistry, Eindhoven University of Technology Eindhoven 5612 AZ the Netherlands S.Li1@tue.nl; e School of Electronic Information and Communications, Huazhong University of Science and Technology Wuhan Hubei 430074 People's Republic of China

## Abstract

Plasma-based CO_2_ conversion is an emerging defossilization technology that converts a potent greenhouse gas into valuable chemical feedstocks, yet its optimization is hampered by complex nonlinear behavior and resource-intensive experimentation. In this work, we collected a comprehensive database, comprising 358 data points with six key operational and geometric parameters, published in literature between 2010 and 2025. Leveraging this dataset, we developed a hybrid machine learning (ML) model integrating physics-informed neural network (PINN), random forest (RF) and extreme gradient boost (XGB) algorithms to predict CO_2_ conversion and energy efficiency (EE) in dielectric barrier discharge (DBD) reactors. Under a rigorous group 5-fold cross-validation (CV) protocol, the ensemble consistently outperformed all individual models, with the best-fold model achieving an *R*^2^ of 0.791. Error-correlation analysis revealed that the ensemble weights adapt to the pairwise error correlation structure: PINN consistently provides complementary information, while RF and XGB, being largely interchangeable, are selected according to their individual performance. When applied to prospective experimental validation, the hybrid model achieves an *R*^2^ of 0.92 on unseen data within the explored domain, and it eliminates unphysical predictions in data-sparse regimes, yielding strictly non-negative CO_2_ conversion estimates. SHapley Additive exPlanations (SHAP) analysis further identified flow rate and power as the dominant input features, collectively accounting for 61%–71% of the model's predictions. This work establishes a robust and interpretable framework while quantifying the generalizability of ML models in heterogeneous data environments, offering a practical tool to accelerate plasma-based gas conversion optimization.

## Abstract 2

A hybrid ML model trained on 358 cross-study data points predicts CO_2_ conversion in DBD reactors with an R2 of 0.92 on unseen experiments, providing an unbiased assessment of model generalizability across heterogeneous experimental sources.

## Graphical abstract

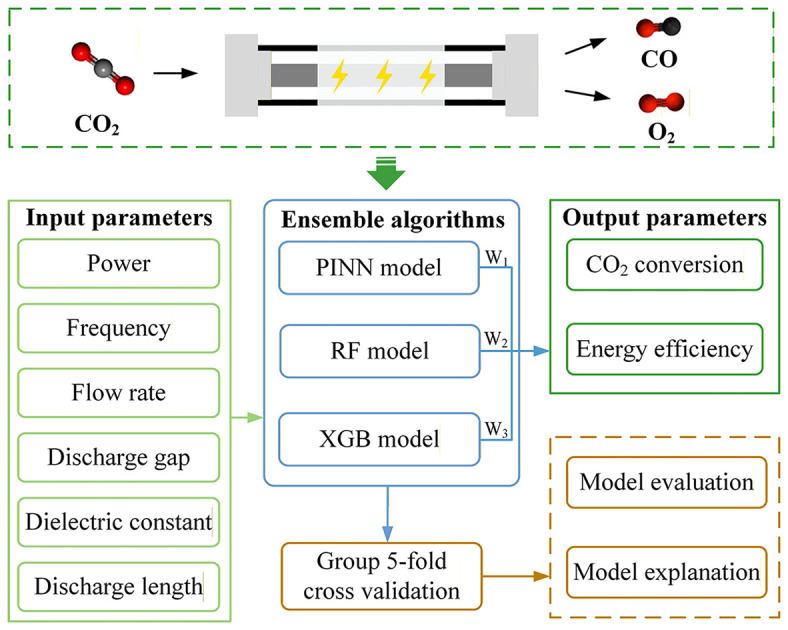

**Green foundation**

1. This work presents a robust and interpretable machine learning (ML) model to predict plasma-based CO_2_ splitting performance from a diverse literature dataset comprising 358 data points, quantitatively assessing model generalizability across heterogeneous experimental sources.

2. Our hybrid model integrating physics informed neural network, random forest and extreme gradient boost algorithms achieves an *R*^2^ of 0.92 on prospective experimental validation while eliminates unphysical predictions on CO_2_ conversion in data sparse regimes.

3. Future studies may focus on advanced ML models development through standardized databases with descriptors including discharge characteristics and when relevant, catalyst properties as well as integration of physics informed architectures to enhance predictive power in plasma-based gas conversion, thereby reducing reliance on extensive experimental campaigns and improving resource efficiency.

## 1. Introduction

Carbon dioxide (CO_2_), a major greenhouse gas driving global climate change, necessitates the urgent development of strategies for its mitigation and valorization. Converting CO_2_ into valuable products is a cornerstone of this effort, yet it is fundamentally challenged by the molecule's high thermodynamic stability, which demands significant energy input for activation. Plasma technology has emerged as a compelling solution to this problem.^1^ By generating a partially ionized gas rich in high-energy electrons, reactive radicals, and excited species, plasma can cleave the stable C=O bond under mild conditions. Notably, non-thermal plasma (NTP) systems, powered by electricity, offer rapid dynamic control and excellent compatibility with intermittent renewable power sources.^2,3^ These advantages position NTP as a key enabling technology for power-to-X (P2X) applications, providing a promising and sustainable pathway for CO_2_ conversion within future energy systems.^4,5^

Various plasma types have been studied for plasma-based CO_2_ conversion.^6,7^ Dielectric barrier discharge (DBD) reactors have attracted particular attention due to their simple design, user-friendly nature, compatibility with catalysts, and potential scalability.^1^ Reported CO_2_ conversion and energy efficiency (EE) values vary widely across studies, even under nominally similar operating conditions, because reactor performance emerges from the coupled effects of discharge power, gas flow rate, electric-field distribution, and reactor geometry.^8,9^ Importantly, these relationships are highly nonlinear and often non-monotonic, with improvements in conversion frequently accompanied by losses in EE. Traditional process optimization approaches in this field rely heavily on computationally expensive physics-based simulations or resource-intensive experiments. Mechanistic modelling, while providing valuable insights,^10,11^ is sometimes constrained by uncertain kinetic parameters or high computational costs for systematic parameter screening. Conversely, purely experimental optimization faces the curse of high dimensionality: the vast parameter space renders exhaustive exploration impractical, possibly leading to suboptimal processes and inefficient resource utilization.^12,13^ This optimization bottleneck constitutes a major barrier, preventing plasma technology from achieving the sustainability performance required for industrial adoption.

Recent advances in machine learning (ML) offer a transformative tool for addressing this challenge.^14–16^ Data-driven algorithms can learn complex input–output relationships directly from experimental observations, enabling rapid performance prediction and rational process optimization without requiring explicit mechanistic knowledge.^17,18^ ML methodologies are typically categorized by their learning model. The most established are supervised learning (SL), which maps known input parameters to target outputs, and unsupervised learning (UL), which identifies latent structures, such as clusters or reduced dimensions, within unlabeled data.^19^ Building on these, more advanced frameworks have emerged: reinforcement learning (RL), where an agent learns optimal actions through environmental feedback and reward signals,^20^ and active learning (AL), designed to maximize model accuracy while minimizing the cost of data annotation.^21^ These powerful data-driven approaches are being increasingly deployed to solve complex problems across scientific domains, including plasma medicine,^22–25^ large-scale screening^26,27^ and synthesis of novel chemicals,^28–30^ self-driving laboratory systems,^31–33^ and emissions control.^34^

ML has also started to be applied in plasma-based gas conversion, specifically in DBD plasmas.^35–38^ Successful applications of several ML models have demonstrated the predictive capability of single-algorithm models, such as artificial neural networks (ANNs). For instance, Wang *et al.* elucidated a relationship between process parameters and performance targets in plasma-based dry reforming of methane (DRM) to oxygenates by well-trained ANN models.^35^ Similarly, Shen *et al.* simulated CO_2_ conversion in a DBD-photocatalytic system using a back-propagation (BP) ANN model, reporting high coefficients of determination (*R*^2^) on both the training set and testing set.^36^ Recently, the field has been rapidly evolving toward more sophisticated frameworks, where pioneering work successfully integrated SL with RL, to not only predict but also autonomously optimize plasma-based or plasma-catalytic CO_2_ and CH_4_ conversion, demonstrating the potential of ML as an active optimization tool.^37,38^ Besides, AL strategies are being employed to automatically and efficiently navigate high-dimensional parameter spaces. For instance, Shao *et al.* used AL *via* Bayesian optimization (BO) to maximize the energy efficiency of NOx generation in a glow discharge within a fixed experimental budget,^21^ while our prior work demonstrated a framework where an ANN, pre-trained on literature data and refined *via* AL with minimal local experiments, achieved *R*^2^ > 0.95 for the prediction performance of the plasma-based CO_2_ splitting.^39^

Despite these promising developments, the research landscape remains nascent and faces significant challenges. Many existing studies rely on small, homogeneous datasets (typically less than 100 data points, which is far from big data and meta-analysis) derived from individual laboratory setups, which limit generalizability. This reliance on group-specific data, coupled with a frequent lack of comprehensive evaluation metrics and model interpretability, creates a “generalizability gap” where high performance reported on one system may not translate reliably to others. To address these limitations, a compelling strategy is to leverage the expanding corpus of published literature to construct larger, more diverse training datasets, fostering the development of widely applicable ML models and enabling robust cross-study comparisons. Recent meta-analysis of CO_2_ conversion data, notably within the PIONEER database framework,^40^ highlights that specific energy input (SEI), *i.e.*, the ratio of power over flow rate, remains the predominant metric for cross-study performance comparison. While practical, this parameter sometimes offers an oversimplified representation, failing to capture the intrinsic and varied plasma properties (*e.g.*, reduced electric field and electron density) within the reaction zone that fundamentally govern the process efficiency. To develop a robust, generalizable ML model capable of accurate cross-experimental prediction, it is therefore essential to move beyond SEI and identify a more comprehensive set of physically grounded descriptors. Moreover, because purely data-driven models lack any inherent awareness of thermodynamic law, they risk producing physically implausible outputs when queried in sparsely sampled operating regimes; incorporating thermodynamic constraints directly into the learning objective offers a paradigm shift from purely empirical fitting toward physically consistent extrapolation.

Furthermore, robust ensemble modelling strategies combined with transparent interpretability frameworks are required. Hybrid ensemble models, which integrate multiple base learners (*e.g.*, neural networks, support vector regression, decision trees or ensemble-tree models), can leverage complementary strengths to enhance predictive robustness and mitigate overfitting, as evidenced in studies on plasma tar reforming and DRM.^41,42^ At the same time, explainable artificial intelligence (XAI) tools are essential for making ML models transparent and interpretable, and help elucidate how a model arrives at its predictions by quantifying the contribution of each input feature to an individual output.^43^ To date, the development of a fully interpretable, physics-informed hybrid ML model, trained exclusively on a comprehensive, multi-source literature dataset for plasma-based CO_2_ conversion has not, to our knowledge, been reported yet.

In this work, we develop a broadly applicable ML framework built on a consolidated literature dataset of 358 experimental conditions for plasma-based CO_2_ splitting in DBD reactors. We systematically evaluate inter-laboratory generalizability through a publication-wise group 5-fold cross-validation strategy, and introduce a physics-informed neural network (PINN) whose loss function is augmented with thermodynamic constraints to prevent unphysical predictions in sparsely sampled operating regimes. The resulting model integrates the PINN with random forest (RF) and extreme gradient boosting (XGB) into a hybrid ensemble, and SHapley Additive exPlanations (SHAP) analysis is employed to quantify feature contributions. This work establishes a robust, interpretable, and thermodynamically consistent ML framework that reduces the reliance on trial-and-error experimentation and provides actionable guidance for process optimization in plasma-based CO_2_ conversion.

## 2. Methods

**Fig. 1:**
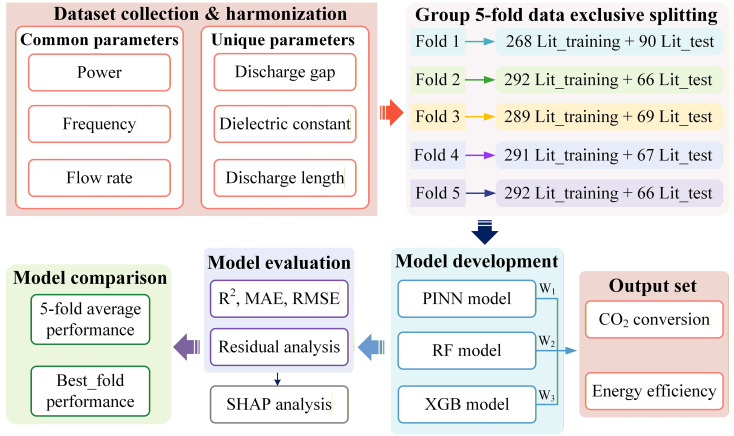
Scheme of the workflow to develop our hybrid ML model.

The framework overview is presented in Fig. 1, with detailed explanations provided in the following sections. A complete list of abbreviations related to these methods is available in the SI (SI, Section S1, Table S1).

### 2.1 Dataset collection and processing

A fundamental challenge in constructing ML models from published literature is the inherent heterogeneity of experimental data, stemming from variations in reactor design, diagnostic methods, and reporting standards. To build a consistent dataset, we executed a rigorous, multi-phase harmonization protocol.

First, a systematic literature screening was conducted, focusing on peer-reviewed studies of CO_2_ splitting in geometrically similar coaxial DBD reactors. The selection of input parameters was guided by the dual principles of widespread data availability and fundamental physical significance. Six key parameters, *i.e.*, discharge power, frequency, gas flow rate, discharge gap, dielectric constant and discharge length, were chosen because they are the most consistently reported descriptors across studies and collectively represent the first-order governing factors of the plasma process: energy input, micro-discharge formation, reactant residence time, electric field strength, charge accumulation and reaction volume, respectively. Consequently, only studies reporting all six inputs alongside measurable CO_2_ conversion and energy efficiency (EE) (or sufficient data for their calculation) were included.

Second, to ensure direct comparability, all performance data were recalculated using unified definitions. CO_2_ conversion was standardized as the molar amount of converted CO_2_ relative to the inlet amount, with corrections for gas expansion where necessary. EE was consistently derived from the discharge power and the enthalpy change of CO_2_ splitting, thereby normalizing values originally reported as SEI or other equivalent metrics (detailed in SI, Section S2). The CO_2_ conversion (*χ*_CO_2__) and EE were defined as follows:^44^1
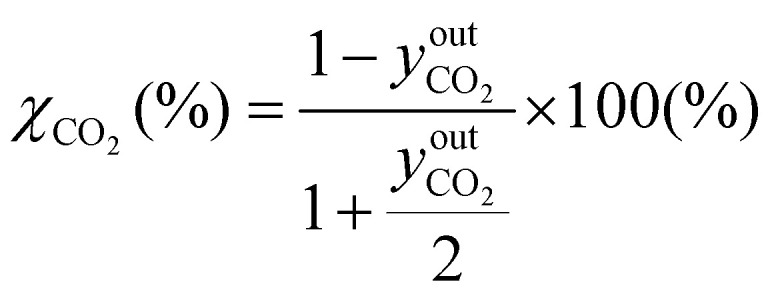
2
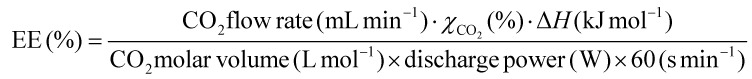
where 
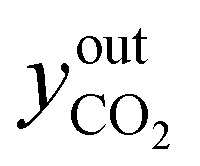
 is the output fraction of CO_2_, Δ*H* is the reaction enthalpy (282.96 kJ mol^−1^) of pure CO_2_ decomposition, and the CO_2_ molar volume is 24.24 L mol^−1^, at around 295 K and 1 atm under ideal gas assumptions.

Finally, this process yielded a compiled dataset of 358 distinct experimental records across 27 published papers,^8,9,39,45–68^ spanning a broad operational range (see Table S2). We explicitly acknowledge that residual, unquantifiable heterogeneity from systematic inter-study differences (*e.g.*, analytical techniques such as GC and FTIR analysis) remains. However, by aggregating data from numerous independent sources, the influence of any single experimental bias is mitigated, allowing the SL model to identify the underlying cross-system trends from a large, diversified dataset. Prior to model training, all features were normalized to a [0, 1] range using the Min–Max scaling technique.

### 2.2 Pearson correlation coefficient

The linear relationship between input parameters and output targets was initially assessed using the Pearson correlation coefficient (PCC). The PCC, which quantifies the strength and direction of a linear relationship, ranges from −1 (a perfect negative correlation) to +1 (a perfect positive correlation).^69,70^ It is calculated as follows:3
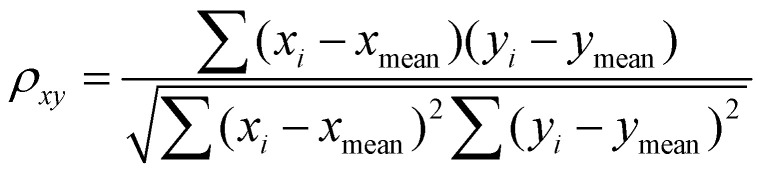
where *ρ*_*xy*_ represents the PCC value between the input feature and output target, *x*_mean_ and *y*_mean_ indicate the average of the input feature *x* and the output target *y*, respectively. For our analysis, the absolute value of the PCC was used to gauge the strength of the linear association between each of the four input parameters and the two target performance metrics (CO_2_ conversion and EE).

### 2.3 Training and validation of ML models

In this study, neural networks (NN) and two tree-based ensemble learning algorithms, namely Random Forest (RF) and Xtreme Gradient Boost (XGB) were first evaluated for their prediction performance. An artificial neural network (ANN) was first constructed as a fully connected, feed-forward architecture with six input neurons (corresponding to discharge power, frequency, gas flow rate, discharge gap, dielectric constant and discharge length) and two output neurons (for CO_2_ conversion and EE). Model training was conducted using the BP algorithm optimized *via* the gradient descent method.^71^ The ReLU activation function was employed in hidden layers to introduce nonlinearity while mitigating the vanishing gradient problem. The mean squared error (MSE) between predictions and experimental values was minimized during training.

Although the standard ANN can accurately capture the complex nonlinear mapping within the training distribution, it lacks any inherent awareness of thermodynamic law. To prevent unphysical predictions when the model is queried outside dense training regions, the ANN was further developed into a physics-informed neural network (PINN) by augmenting the loss function with a penalty term that enforces fundamental thermodynamic constraints on the predicted CO_2_ conversion (*X*_pred_, in %) and EE (EE_pred_, in %):4
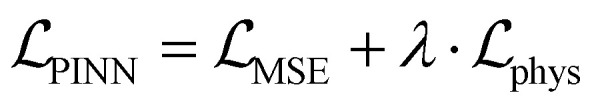
with5
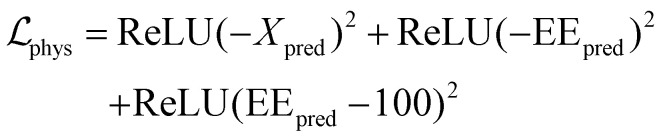
where the penalty factor *λ* was set to 10^−3^. The three terms enforce non-negative conversion, non-negative EE, and EE not exceeding 100%, respectively. The first two terms enforce non-negativity of the predicted conversion and energy efficiency. The last constraint, EE_pred_ ≤ 100%, is equivalent to the thermodynamic upper bound imposed by the minimum enthalpy of CO_2_ dissociation. This equivalence follows directly from the definition of EE in eqn (2): since EE ∝ *χ*_CO_2__/(P/F), a predicted EE exceeding 100% would imply a conversion greater than the theoretical maximum attainable for the given power and flow rate, thereby requiring less than the reaction enthalpy of 282.96 kJ per mole of CO_2_ split. Apart from the modified loss function, all other training settings remained identical to the standard ANN.

RF is an ensemble method that operates on the principle of bootstrap aggregation, or bagging.^72^ It constructs a large collection of decorrelated decision trees, each trained on a random subset of the data and features. For regression, the final prediction is formed by averaging the output of all individual trees in the forest. This methodology effectively reduces model variance, mitigates overfitting, and enhances generalization performance compared to a single decision tree, yielding a robust predictive model.

XGB is a highly efficient and scalable implementation of the gradient boosting framework.^70^ It builds decision trees sequentially, with each new tree trained to correct the residual errors of the current ensemble. The algorithm incorporates advanced regularization techniques to control model complexity and prevent overfitting. Key computational innovations, such as a weighted quantile sketch for efficient candidate split finding, enable faster training and often superior accuracy compared to other tree-based models like RF.

To obtain a realistic estimate of model generalizability across different published studies and to avoid data leakage, we adopted a group 5-fold cross-validation (CV) strategy with the source publication as the grouping variable. The 27 source publications were partitioned into five folds such that each fold was held out for testing exactly once across the five folds and used for training in the remaining four folds, thereby providing an unbiased assessment of the inter-laboratory and inter-publication predictive performance. Because the number of data points varies among source publications, the group assignment was designed to keep the validation set size in each fold at approximately 20%–25% of the total dataset, balancing the evaluation across folds while strictly preserving group integrity.

Hyperparameter optimization for all algorithms was conducted using the BO method (see Section S3, SI),^36^ which efficiently explores high-dimensional parameter spaces by constructing probabilistic surrogate models (Gaussian processes) that guide the search toward optimal configurations with minimal computational cost. In principle, a single set of hyperparameters could be selected to maximize the average performance across the five folds. However, the considerable heterogeneous dataset employed in this study comprises multiple literature sources with widely varying value ranges in each fold, a fixed hyperparameter configuration that performs well on one fold frequently performs poorly on another, occasionally driving the overall five-fold average into a negative coefficient of determination (*R*^2^) regime. We therefore performed independent hyperparameter optimization within each of the five mutually exclusive splits, yielding five separate model instances.

### 2.4 Hybrid model development and evaluation

To integrate the complementary strengths of the individual base learners, a hybrid ensemble model was constructed. This model generates its final prediction through a linear combination of the predictions from the three evaluated algorithms, namely, PINN, RF, and XGB, as defined by the following eqn (6):6
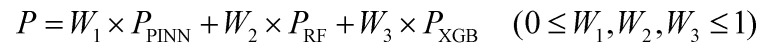


Following the independent hyperparameter optimization of the base learners within each fold, the optimal ensemble weights were determined *via* an exhaustive grid search over the interval [0, 1] with a step size of 0.01. For each candidate weight combination, the ensemble prediction was constructed as a linear combination of the three base model outputs, and *R*^2^ on the corresponding validation set was computed. The weights that simultaneously maximized *R*^2^ were selected for that fold. This procedure was repeated independently for each of the five folds, yielding five-fold-specific ensembles. The reported CV metrics represent the average performance of these five independently optimized ensembles on their respective validation folds.

To comprehensively evaluate the model's performance, several metrics, including *R*^2^, MSE, mean-absolute error (MAE), and root mean square error (RMSE), were computed to test the datasets and validate the robustness and generalizability, as shown in Table 1.

**Table 1:** Metrics used for evaluating the performance of the ML models

Metric	Definition	Equation
Coefficient of determination (*R*^2^)	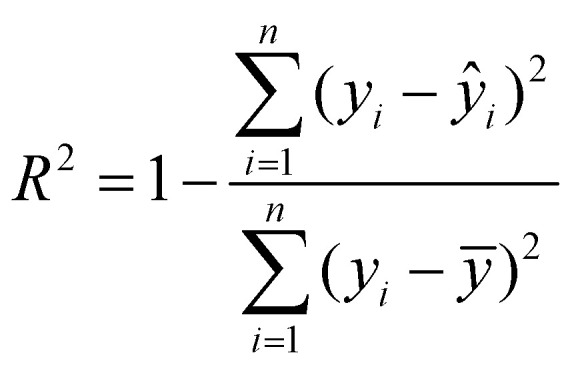	(7)
Mean-squared error (MSE)	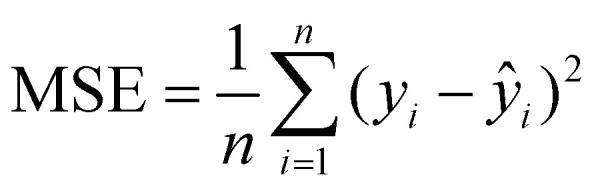	(8)
Mean-absolute error (MAE)	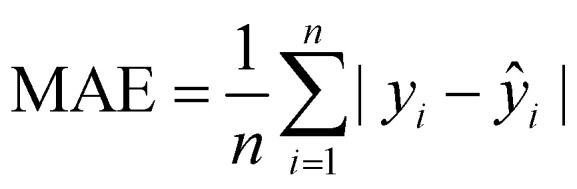	(9)
Root mean square error (RMSE)	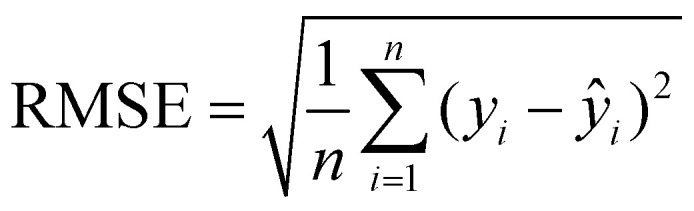	(10)

### 2.5 SHapley Additive exPlanations (SHAP) methodology

SHAP provides a model-agnostic framework for interpreting ML predictions, grounded in concepts from cooperative game theory. At its core, SHAP quantifies the contribution of each input feature to a model's output by calculating its Shapley value. This value represents the average marginal contribution of the feature to the prediction, considering all possible combinations of other input features. The Shapley value for a feature is given by:^73^11
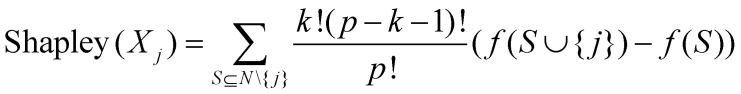
where *p* is the total number of input features, *N*\{*j*} is the set of all features excluding *X*_*j*_, and *S* denotes a specific subset from *N*\{*j*}. The terms *f*(*S*) and *f*(*S*∪{*j*}) correspond to the model's predictions using the feature subset *S* and when feature *X*_*j*_ is added to *S*, respectively.

A key strength of the SHAP framework is its dual capacity for interpretation. It offers local interpretability by assigning a precise contribution value to each feature for every individual prediction. Simultaneously, global interpretability is achieved by aggregating absolute Shapley values across the entire dataset, which reveals the overall average influence of each feature on the model's outputs.^74,75^ A higher absolute mean SHAP value indicates a feature with greater overall influence on the model's predictions. It is essential to emphasize a critical distinction: SHAP analysis explains the model's behavior, not the underlying physical system. The method identifies which input features the trained model found most statistically influential for its predictions. It does not establish causal relationships, nor does it elucidate the fundamental mechanistic role of parameters within the plasma-chemical process. Therefore, SHAP values indicate “what the model relies on”, providing essential insight into its decision-making process, which must then be reconciled with domain knowledge.

## 3. Results

### 3.1 Characterization of the training dataset

The statistical summary of the compiled and harmonized dataset, presented in Table 2, provides a clear overview of the operational landscape captured from the literature. The parameter ranges are extensive, reflecting the broad exploration conducted by the research community. Discharge power varies over three orders of magnitude, from 0.5 W to 1000 W. Gas flow rate spans an even wider range, from 10 to 3000 mL min^−1^. Discharge gap and length also cover significant ranges, from sub-millimeter scales to several centimeters. Additionally, the frequency ranges from 0.05 kHz to 120 kHz, and the dielectric constant spans from 3.7 to 10. This breadth is beneficial for model training, as it exposes the algorithms to a wide spectrum of reactor behaviors and provides leverage to learn non-linear trends.

**Table 2:** Statistical summary of the compiled literature dataset

Parameter	Minimum	Maximum	Mean	Std. deviation
Power (W)	0.5	1000	42.25	68.84
Flow rate (mL min^−1^)	10	3000	86.96	210.35
Frequency (kHz)	0.05	120	23.87	19.67
Discharge gap (mm)	0.25	8.00	2.09	1.66
Length (cm)	1.0	40	11.73	7.25
Dielectric constant	3.7	10	6.76	2.85
CO_2_ conversion (%)	1.06	54.49	17.08	10.55
Energy efficiency (%)	0.55	23.34	5.11	4.06

At the same time, the data density is not uniform across these ranges; conditions around moderate flow rates (25–100 mL min^−1^) and mid-range powers (20–50 W) are more densely sampled, reflecting common laboratory practice. This non-uniform coverage is an important consideration when interpreting model uncertainty and generalization performance. The output variables (CO_2_ conversion and EE) exhibit correspondingly wide distributions, ranging from below 2% to over 50% for conversion and from under 1% to over 20% for efficiency, capturing the diversity of performance outcomes across different reactor configurations and operating conditions. This non-uniform coverage is an important consideration when interpreting model uncertainty and generalization performance.

### 3.2 Relative importance analysis

**Fig. 2:**
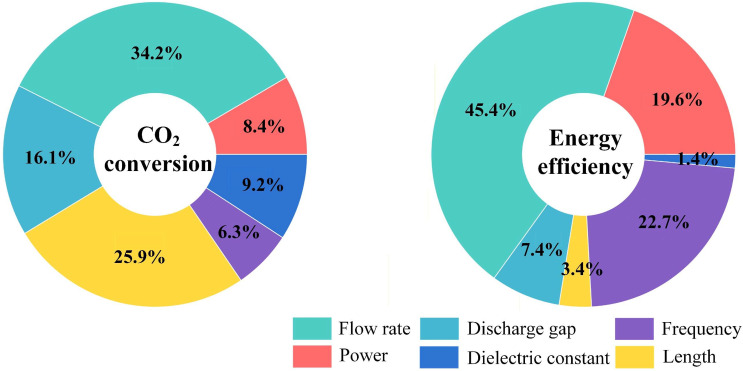
Relative significance of operating parameters on the performance of plasma-based CO_2_ splitting.

The relative importance of each input parameter, as quantified by Pearson correlation coefficient (PCC) analysis, is demonstrated in Fig. 2. Flow rate emerged as the most important input parameter for both CO_2_ conversion (34.2%) and energy efficiency (45.4%). Frequency and power are the second- and third-most important parameters for EE (22.7% and 19.6%, respectively), while being the least important parameters for CO_2_ conversion (6.3% and 8.4%, respectively). In contrast, discharge length and gap are the second and third most important parameters for CO_2_ conversion (25.9% and 16.1%, respectively) while ranking among the least important for EE (3.4% and 7.4%, respectively). Dielectric constant exhibits moderate importance for CO_2_ conversion (9.2%, fourth) but negligible influence on EE (1.4%).

### 3.3 Performance of individual ML models

**Fig. 3:**
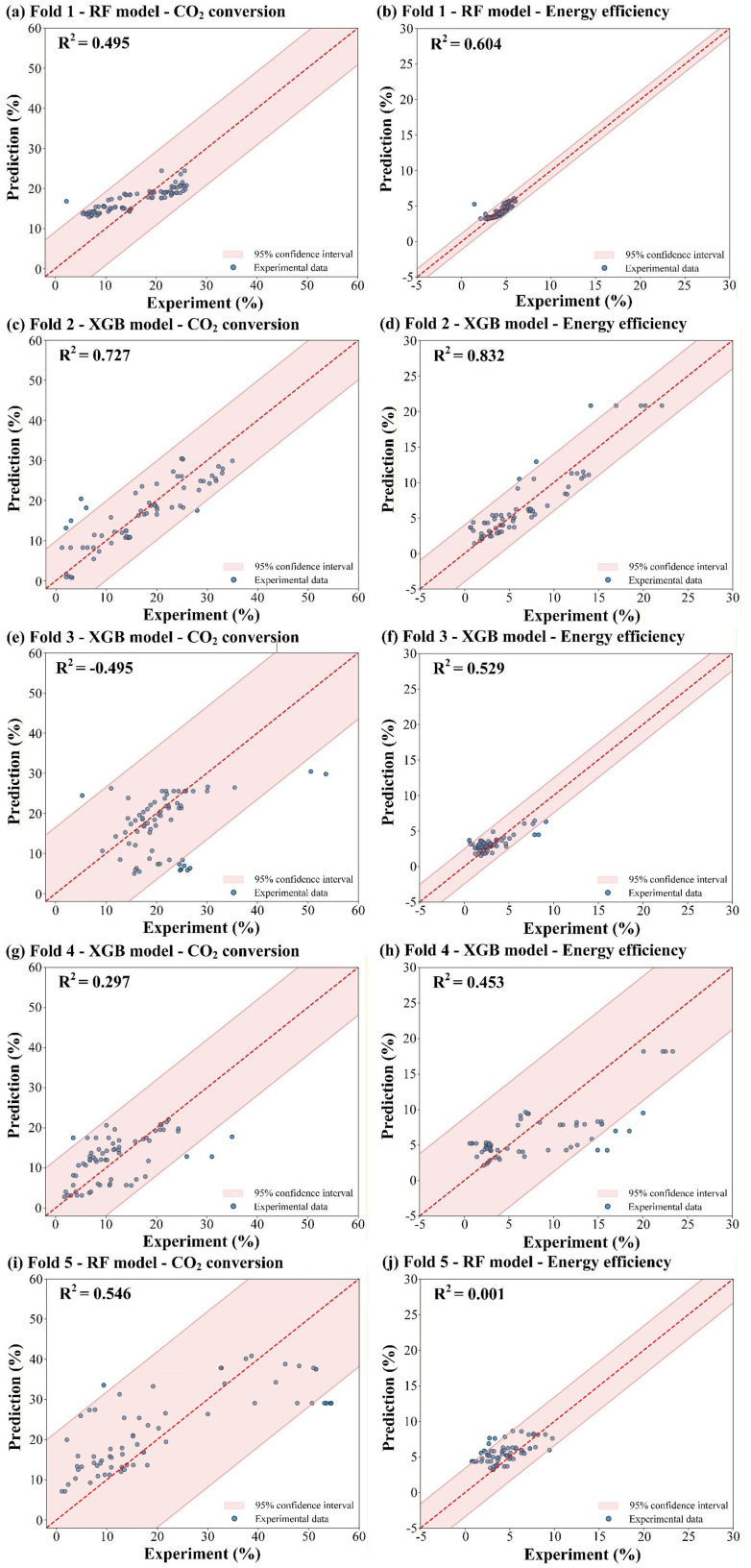
Predicted data *versus* experimental results on the dataset (*R*^2^ plot) for the single optimal model for CO_2_ conversion and energy efficiency within the (a, b) Fold 1, (c, d) Fold 2, (e, f) Fold 3, (g, h) Fold 4, and (i, j) Fold 5.

To construct an efficient ML model, benchmark tests were first conducted on individual ML models, namely PINN, RF, and XGB. Hyperparameters and training curves of these models are shown in Table S3 and Fig. S1 in the SI, respectively. The predictive performance of the three base learners under the group 5-fold CV framework is summarized in Table 3. The XGB model achieved the highest five-fold average *R*^2^ of 0.362 and the lowest error metrics overall, followed by the RF model. PINN exhibited the weakest overall performance, with an average *R*^2^ of 0.038 and the highest error metrics. However, the best individual model varied across folds. RF outperformed the XGB model in Folds 1 and 5, while the XGB model was the strongest learner in Folds 2, 3, and 4, as shown in Fig. 3.

**Table 3:** Overall performance of individual ML models within the group 5-fold cross-validation framework

Fold	Model	*R* ^2^	MSE	RMSE	MAE
Fold 1	PINN	0.055	28.704	4.152	3.659
	RF	0.549	12.116	2.735	2.306
	XGB	0.417	10.669	2.670	2.257
Fold 2	PINN	0.660	24.162	4.419	3.488
	RF	0.740	17.766	3.843	2.992
	XGB	0.780	14.590	3.518	2.739
Fold 3	PINN	−1.077	29.387	5.127	4.009
	RF	−0.658	47.864	5.948	4.803
	XGB	0.017	42.336	5.193	3.645
Fold 4	PINN	0.288	34.843	5.618	4.433
	RF	0.325	31.293	5.555	4.502
	XGB	0.375	29.106	5.348	4.088
Fold 5	PINN	0.267	89.789	7.586	5.871
	RF	0.273	64.598	6.635	5.085
	XGB	0.220	77.236	7.181	5.587
Group 5-fold average	PINN	0.038	41.377	5.380	4.292
	RF	0.246	34.727	4.943	3.938
	XGB	0.362	34.787	4.782	3.663

Substantial inter-fold variability was observed across all models, reflecting the heterogeneous nature of the multi-source dataset and the strict source-holdout partitioning. In Fold 2, all three models performed well, with the XGB model reaching an *R*^2^ of 0.780, RF attaining 0.740, and PINN achieving 0.660. In Fold 3, however, performance collapsed across the board: PINN and RF returned sharply negative *R*^2^ values of −1.077 and −0.658, respectively, while XGB was only marginally positive (*R*^2^ = 0.017). Folds 1 and 4 showed intermediate performance, with the best model achieving *R*^2^ values of 0.549 and 0.375, respectively. PINN displayed the widest performance swing, underscoring its sensitivity to the composition of the training and test sources. These results demonstrate that while XGB provides the most consistent baseline overall, no single model is immune to the distribution shifts inherent in strict group-wise validation.

### 3.4 Performance of hybrid ML models

**Fig. 4:**
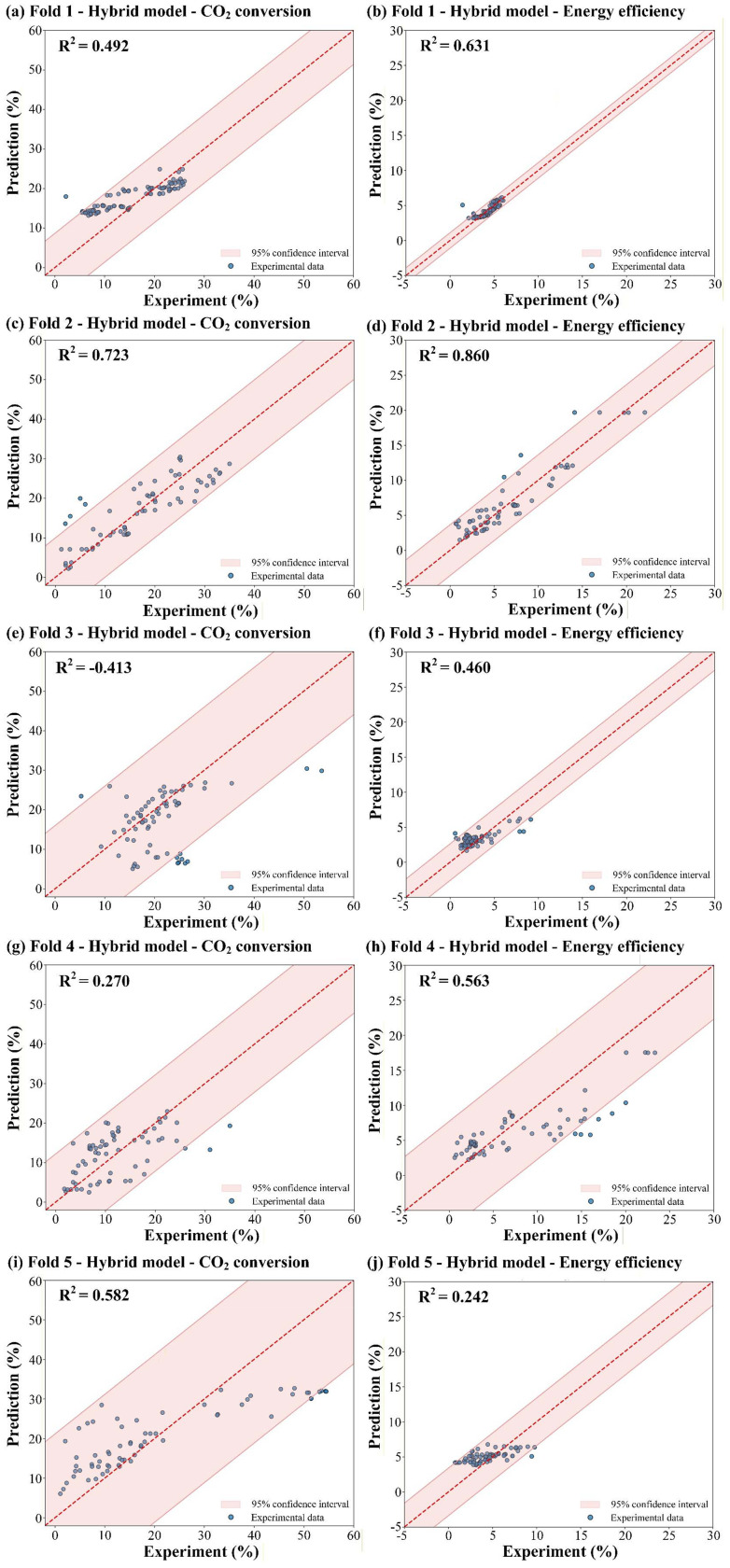
Predicted data *versus* experimental results on the dataset (*R*^2^ plot) for the hybrid model for CO_2_ conversion and energy efficiency within the (a, b) Fold 1, (c, d) Fold 2, (e, f) Fold 3, (g, h) Fold 4, and (i, j) Fold 5.

The hybrid ensemble model was then evaluated under the same group 5-fold CV framework, and its performance in each fold is summarized in Table 4 and Fig. 4. The ensemble consistently surpassed the best individual model in every fold, achieving a five-fold average *R*^2^ of 0.441, a 21.8% improvement over the strongest single learner (XGB, average *R*^2^ of 0.362), alongside corresponding reductions in all error metrics.

**Table 4:** Overall performance of the hybrid ML models within the group 5-fold cross-validation framework

Models	Weights (PINN/RF/XGB)	*R* ^2^	MSE	MAE	RMSE
Fold 1	0.13/0.87/0.00	0.562	12.172	2.238	2.731
Fold 2	0.21/0.06/0.73	0.791	14.441	2.618	3.449
Fold 3	0.07/0.00/0.93	0.024	40.162	3.581	5.110
Fold 4	0.36/0.02/0.62	0.417	27.665	4.077	5.161
Fold 5	0.47/0.25/0.28	0.412	59.065	4.803	6.271
5-Fold average	—	0.441	30.701	3.463	4.544

**Fig. 5:**
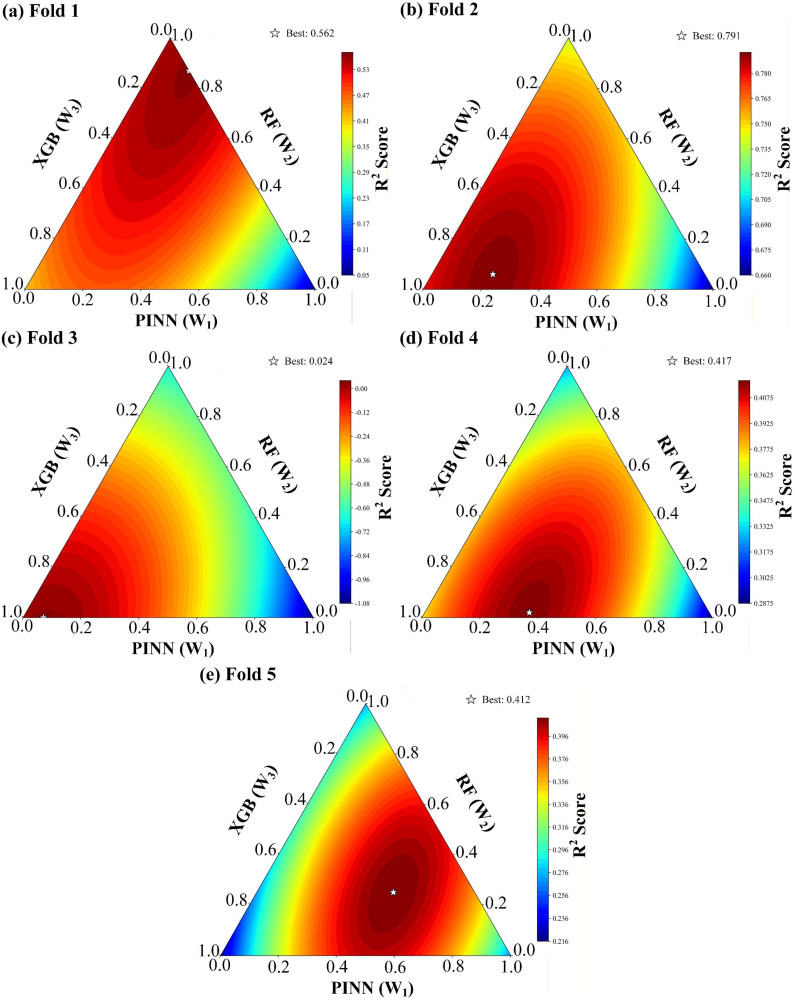
*R* ^2^ of the training and optimization process for the hybrid model within (a) Fold 1, (b) Fold 2, (c) Fold 3, (d) Fold 4 and (e) Fold 5.

The optimized weights varied considerably across folds, reflecting the adaptive nature of the fusion strategy that responded to the characteristics of each held-out source, as shown in Fig. 5. In most folds, the ensemble converged to a two-model configuration: either RF was excluded entirely (Folds 2 and 4) or dominated the combination (Fold 1), while XGB received the largest share in three of the five partitions. A notable exception occurred in Fold 5, where all three base learners contributed meaningfully (0.47, 0.25, and 0.28 for PINN, RF, and XGB, respectively), which also delivered the largest gain over the best single model in that fold. In the most challenging partition (Fold 3), the ensemble concentrated 0.93 of the weight on XGB, the only model with a positive individual *R*^2^, yielding a marginally positive ensemble *R*^2^ of 0.024. The broad dispersion of optimal weights across folds confirms that no fixed weighting scheme is universally effective, and that the ensemble's strength lies precisely in its ability to rebalance model contributions according to the difficulty and composition of each held-out source.

**Fig. 6:**
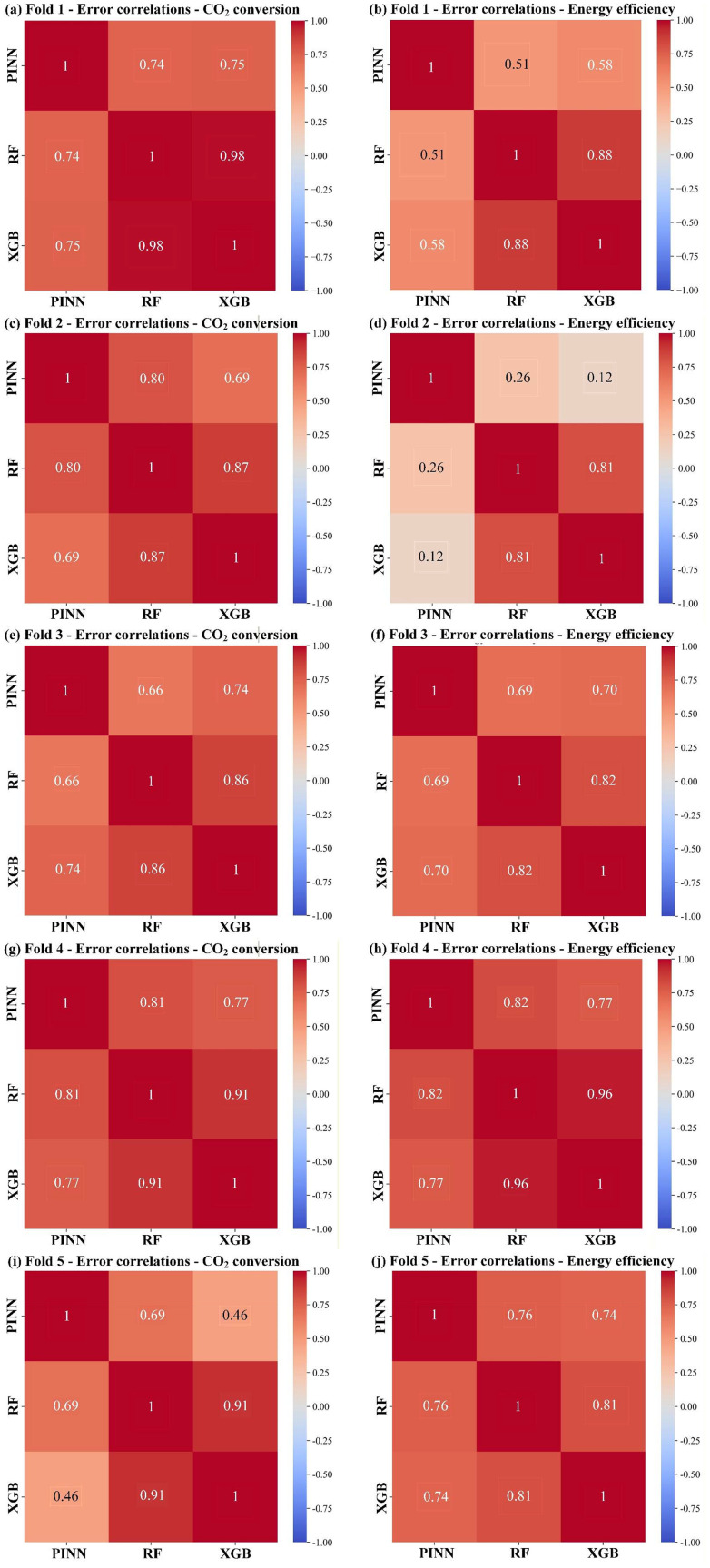
Error complementarity of the PINN, RF and XGB models for CO_2_ conversion and energy efficiency for CO_2_ conversion and energy efficiency within the (a, b) Fold 1, (c, d) Fold 2, (e, f) Fold 3, (g, h) Fold 4, and (i, j) Fold 5.

The pairwise error correlations among the three base learners were examined in each fold to characterize the diversity of their prediction errors. We computed the PCC of their prediction residuals on the different validation fold sets, as shown in Fig. 6. Across all folds, the error correlation between RF and XGB almost consistently exceeds 0.8, indicating that the two tree-based models tend to make similar mistakes. The correlation between PINN and the tree-based models showed greater variation across folds. In Folds 1, 3, and 4, PINN-RF and PINN-XGB correlations were broadly comparable, generally ranging from 0.51 to 0.82. In Fold 2, a notable asymmetry appeared for EE, where PINN-RF and PINN-XGB correlations were considerably lower (0.26 and 0.12, respectively). Fold 5 displayed a distinct structure, with PINN maintaining moderate correlations with RF (0.69 and 0.76) and noticeably lower correlations with the XGB model for CO_2_ conversion (0.46), representing the lowest pairwise correlation with the XGB model observed across all folds.

Overall, while the RF and XGB models consistently produced highly correlated errors, PINN exhibited a systematically lower, albeit variable, degree of correlation with both, reflecting its architectural distinctness. This error correlation structure provides the basis for the weight optimization to selectively engage complementary models in each partition (see Discussion 4.2).

**Fig. 7:**
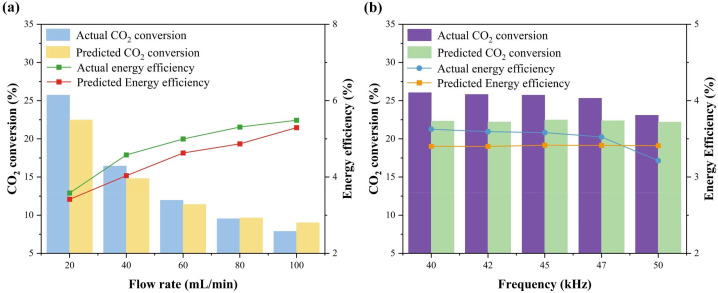
Predicted performance (in terms of CO_2_ conversion and EE) by the hybrid model for unseen experimental datasets, compared with the actual data, as a function of (a) flow rate (power = 27.93 W, frequency = 45 kHz and discharge gap = 1.05 mm) and (b) frequency (flow rate = 20 mL min^−1^ and discharge gap = 0.8 mm). Discharge power, plasma length and dielectric constant are fixed at 27.93 W, 7.5 mm and 9.6, respectively.

To assess the generalizability of the hybrid model, we conducted validation experiments at operating conditions within the training parameter ranges but absent from the literature database. Fig. S2 in the SI shows the experimental setup of the DBD reactor used for the CO_2_ splitting experiments, described in detail in ref. 76. The predictions of the hybrid model for the unseen data exhibit excellent agreement with the experimental results, as presented in Fig. 7. The model predictions closely track the experimental trends for both CO_2_ conversion and EE, as a function of either flow rate or frequency, achieving an *R*^2^ of 0.924. This strong performance on truly independent, prospective experimental data confirms the model's reliability and practical utility for guiding reactor design and operational optimization.

### 3.5 Extrapolation capability of the PINN model

To evaluate the impact of physics-informed training, the PINN was compared against a purely data-driven ANN with an identical training procedure but without the thermodynamic penalty on Fold 2, the most favorable partition. Its architecture was independently optimized *via* BO under the standard MSE loss, yielding a different hidden-layer configuration. Despite this architectural freedom, the standard ANN achieved a markedly lower individual *R*^2^ than the PINN, and when integrated into the ensemble, it received zero weight and was thus entirely excluded. The PINN, by contrast, retained a meaningful complementary role within the ensemble (Table 5).

**Table 5:** Comparison of the performance of the PINN and ANN models on Fold 2

Key feature	PINN	ANN
Neurons in hidden layers	(45, 38, 20)	(45, 13, 13)
Loss function	MSE + *λ* × loss_phys	MSE
Weights (NN, RF, XGB)	(0.21, 0.06, 0.73)	(0.00, 0.23, 0.77)
NN *R*^2^	0.660	0.403
Hybrid *R*^2^	0.791	0.783

To delineate the predictive envelope of the ensemble models, we examined relatively extreme operating conditions (*e.g.*, high flow rate of 1000 mL min^−1^, power between 18 and 38 W) that lie near the boundary of the training distribution but are sparsely represented in the collected literature. In the entire dataset, high-flow conditions appear almost exclusively at power levels above 50 W, leaving the low-power, high-flow quadrant virtually unexplored. Table 6 compares the predictions of the individual ANN and the hybrid PINN model on five extrapolated conditions.

**Table 6:** Comparisons of the predictions of the two model variants on extrapolated conditions

Flow rate (mL min^−1^)	Power (W)	Actual CO_2_ conversion (%)	Actual EE (%)	ANN prediction		Hybrid PINN prediction	
				Conversion (%)	EE (%)	Conversion (%)	EE (%)
1000	18.68	0.41	4.27	−1.06	33.89	0.66	11.70
1000	24.74	0.68	5.35	−1.16	33.79	0.71	11.78
1000	27.93	0.61	4.25	−1.20	33.73	0.74	11.70
1000	31.83	0.75	4.58	−1.26	33.67	0.77	11.59
1000	37.89	0.94	4.82	−1.35	33.56	0.81	11.62

The standard ANN produces negative CO_2_ conversion values across all five test points, while simultaneously overestimating energy efficiency by roughly a factor of seven. This failure occurs because the network extrapolates a decreasing conversion trend linearly across zero when confronted with the unseen combination of high flow and low power. The hybrid PINN model, however, eliminates all negative predictions entirely, returning strictly positive conversion values that approach the measured range, and reduces the EE overestimation to approximately a factor of two to three. While the quantitative accuracy of the PINN-based hybrid in this extreme extrapolation regime remains imperfect, the principal achievement is the complete removal of the catastrophic unphysical predictions that characterized the unconstrained model.

### 3.6 Model interpretability: SHAP analysis

**Fig. 8:**
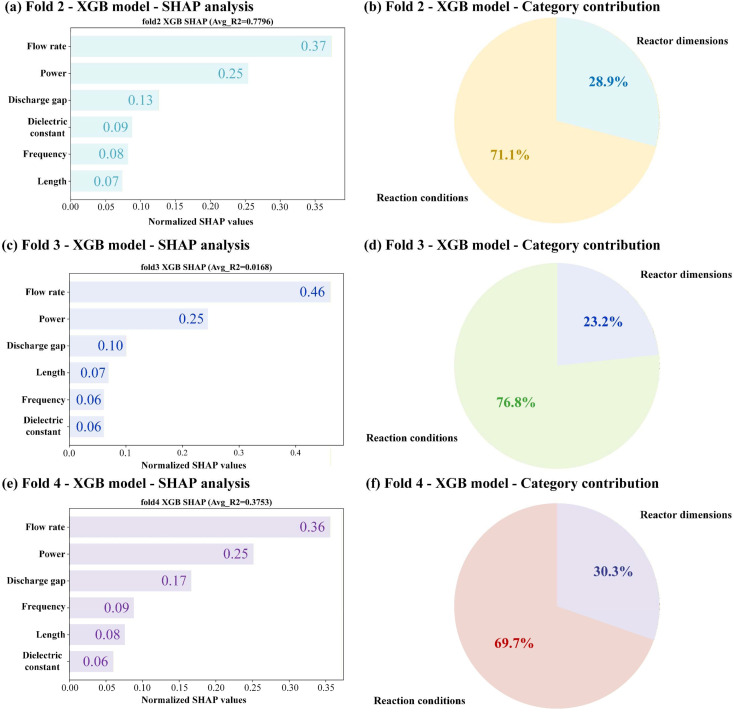
Feature importance analysis represented in the form of horizontal bar plots for normalized SHAP values, and a pie chart visualizing the cumulative contribution of the reaction conditions and reactor dimensions by the model prediction in (a, b) Fold 2, (c, d) Fold 3 and (e, f) Fold 4.

SHAP analysis was performed on the best XGB model component of the hybrid model across Fold 2, 3 and 4 to interrogate the contribution of individual input features and to assess the stability of the derived interpretability metrics, as shown in Fig. 8. The six input descriptors encompass two distinct category domains: reaction conditions (flow rate, power, and frequency) and reactor dimensions (discharge gap, dielectric constant, and length). At the level of individual features, a clear and reproducible hierarchy emerged. Flow rate consistently ranked as the most influential descriptor across all three folds, followed by power, with discharge gap occupying the third position. The relative ordering of these top three features remained invariant across folds, while the lower-ranked features, *i.e.*, dielectric constant, frequency, and length, exhibited minor positional fluctuations that did not alter the overall importance structure.

In global interpretability analysis, the reaction conditions dominated the reactor dimensions by a factor of approximately two across all folds examined: the share of reaction conditions ranged from 69.7% (Fold 4) to 76.8% (Fold 3), while reactor dimensions accounted for the complementary fraction. The constancy of this categorical imbalance across different ensemble weight configurations confirms that the feature importance ranking reflects genuine data structure rather than model-specific artifacts. It is important to emphasize that ensemble weights and SHAP values address fundamentally different questions. Ensemble weights reflect the optimal proportion for combining predictions from different algorithms to minimize overall error. In contrast, SHAP values decompose the prediction of the already-weighted hybrid model to attribute credit to each input feature. Thus, ensemble weights pertain to the model combination strategy, while SHAP values explain feature contributions within the combined model. These two types of “importance” are not directly comparable and should not be conflated.

## 4. Discussion

### 4.1 Relative significance and model interpretability

The PCC identifies gas flow rate as the most statistically significant parameter for both CO_2_ conversion and EE. Flow rate governs critical process variables, such as reactant residence time, species concentration within the discharge zone, and the resulting thermal and chemical history of the gas mixture.^77^ The dominant role of flow rate stems from its direct control over two competing physical factors: residence time and SEI. A lower flow rate increases residence time, enhancing conversion, but also raises the SEI, which is detrimental to EE. Conversely, a higher flow rate typically benefits EE by lowering SEI, but limits conversion due to reduced residence time. Input power shows a strong correlation with EE as it is the numerator in the SEI. Its relatively weak correlation with conversion indicates that in the studied regime, increasing power primarily leads to gas heating and acceleration of recombination reactions rather than to net conversion gains. Therefore, optimizing for high efficiency requires carefully balancing a sufficiently high flow rate to maintain a low optimal SEI with just enough power to achieve the desired conversion level, avoiding the diminishing returns associated with excessive power input.^78^

The reactor geometry, specifically a smaller discharge gap and a longer discharge length, is statistically significant for enhancing CO_2_ conversion but shows limited correlation with EE. This can be explained by the underlying plasma physics: a smaller gap increases the reduced electric field strength, promoting more efficient electron-impact dissociation of CO_2_,^55,79^ while a longer discharge length increases reactant residence time within the plasma zone.^55^ However, these geometric improvements do not directly address the fundamental limitation of the back-reaction between CO and O_2_, which wastes input energy and creates a known trade-off between conversion and EE.^46^ Therefore, while geometry is a key lever for conversion, breaking the conversion-efficiency trade-off likely requires advanced strategies such as *in situ* product separation or plasma-catalyst synergy.^46,80^

Regarding model interpretability, it is critical to recognize that SHAP values reflect conditional associations learned from the training data distribution and do not provide direct evidence of independent physical causality by which these parameters affect plasma chemistry. Two statistical properties of the dataset govern the observed feature rankings: differential variance and multivariate collinearity. First, the high SHAP magnitudes assigned to flow rate and power (collectively 61%–71%) are partially attributable to their broader sampling ranges relative to other descriptors. Tree-based models preferentially split on high-variance features; thus, importance rankings are shaped by experimental design rather than intrinsic physical dominance alone. Second, plasma process variables are inherently collinear. Flow rate and power are coupled through the derived quantity SEI, a central parameter governing EE in plasma processes. In typical experimental designs, power and flow rate are adjusted in tandem to systematically explore the SEI space, creating a structured correlation between these two input features. At constant SEI, the power and flow rate vary proportionally; across the dataset, their variations are linked through this ratio. Consequently, the model cannot independently resolve the distinct physical roles of flow rate and power because their effects are statistically entangled *via* SEI. SHAP attribution under such collinearity is conditional on the covariance structure of the training data and cannot isolate independent mechanistic effects.

Therefore, the SHAP-derived rankings should be interpreted strictly as a descriptive summary of the model's internal association structure within the sampled parameter space. They do not validate that the model has learned physically meaningful parameters, nor are they transferable to regimes with different covariance structures. Disentangling the independent contributions of collinear variables would require causally informed experimental designs that deliberately decorrelate power and flow rate.

### 4.2 Impact of validation strategy on performance assessment

**Fig. 9:**
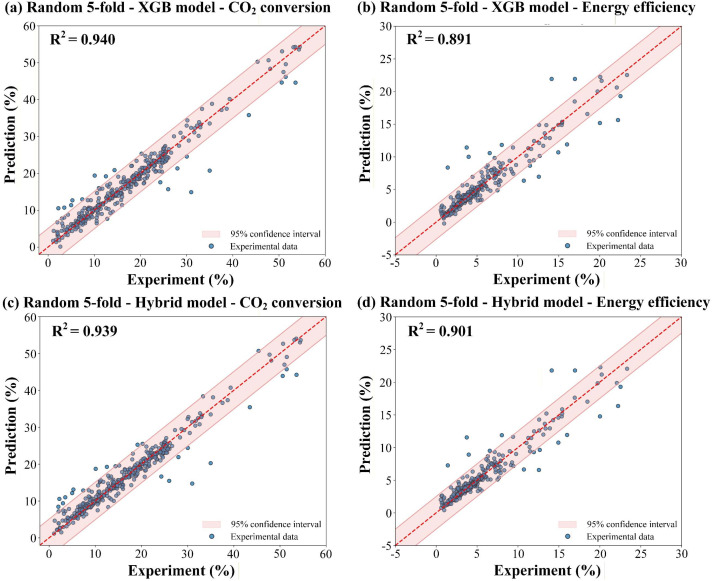
Predicted data *versus* experimental results on the dataset (*R*^2^ plot) within the random 5-fold cross-validation framework for the XGB model for (a) CO_2_ conversion and (b) energy efficiency, and hybrid model for (c) CO_2_ conversion and (d) energy efficiency.

To evaluate the influence of the validation strategy, we compared the XGB and hybrid models under group 5-fold CV and standard randomized 5-fold CV, as presented in Fig. 9. The hyperparameters of the randomized 5-fold CV model are shown in Table S4 in the SI. Under randomized splitting, both the XGB and hybrid model achieved average *R*^2^ values above 0.91, more than double the corresponding Group CV results (*R*^2^ = 0.441), and even exceeded the performance observed in the most favorable Group CV fold (*R*^2^ = 0.791). This pronounced disparity quantifies the severe inflation introduced by data leakage when interdependent points from the same published study are permitted to span both training and validation sets. The contrast reveals that conventional random splitting can yield deceptively optimistic assessments that collapse under genuine cross-study deployment, underscoring the necessity of group-aware validation for any ML model trained on aggregated literature data.

The performance gap between these two frameworks exemplifies a fundamental tension in applying ML to plasma-based CO_2_ conversion: the conflict between model expressiveness, dataset heterogeneity, and evaluation authenticity. The modest *R*^2^ obtained under Group CV is not a failure of the modelling approach, but an honest quantification of how much predictive power can be transferred across laboratories with distinct reactor designs, diagnostic methods, and measurement protocols. The inflated metrics under randomized splitting, by contrast, reveal only that the model can interpolate effectively within a consistent data distribution between training and testing, a capability that is of limited practical value when the goal is to predict performance in an unseen experimental setup.

In summary, these findings highlight a key principle for sustainable process development: systematic investment in standardized, high-quality data collection yields compounding returns in model reliability and transferability. As consistent data accumulate, generalization performance can progressively approach the idealized accuracy, ultimately reducing the collective experimental burden for plasma process optimization across the research community.

### 4.3 Performance and applicability of the weighted ensemble models

Table 7 summarizes the error correlation for reaction performance, weight assignment and corresponding model performance. The ensemble weight distributions observed across the five Group CV folds fall into three archetypal modes, jointly governed by the pairwise error correlations among the base models and their individual predictive power. Across all folds, RF and XGB exhibit high error correlation, making the two tree-based models largely interchangeable, whereas PINN maintains low to moderate correlations with both, providing a persistent source of complementary information. Consequently, when one tree-based model substantially outperforms the other, the weaker one is suppressed to near-zero weight to form a two-active-model configuration, as seen in Fold 2 and 4, where XGB dominated, and RF was excluded, and in Fold 1, where the reverse occurred. PINN is almost always retained with a non-zero weight because its distinct architecture supplies error patterns not captured by the tree-based models. The only extreme case is Fold 3, where PINN joined RF in returning a negative *R*^2^ far below that of XGB, and the ensemble degenerated to XGB alone. A genuine three-model configuration emerged only in Fold 5, where all three learners performed comparably, and PINN maintains only moderate error correlation, with both tree-based models allowed to contribute meaningful complementary information.

**Table 7:** Error correlation for reaction performance, weight assignment and corresponding model performance

Fold	Error correlation for CO_2_ conversion			Error correlation for EE			Weights (PINN/RF/XGB)	Optimal single model_*R*^2^	Hybrid model_*R*^2^	Δ*R*^2^
	PINN-RF	PINN-XGB	RF-XGB	PINN-RF	PINN-XGB	RF-XGB				
1	0.74	0.75	0.98	0.51	0.58	0.88	0.13/0.87/0.00	0.549 (RF)	0.562	0.013
2	0.80	0.69	0.87	0.26	0.12	0.81	0.21/0.06/0.73	0.780 (XGB)	0.791	0.011
3	0.66	0.74	0.86	0.69	0.70	0.82	0.07/0.00/0.93	0.017 (XGB)	0.024	0.007
4	0.81	0.77	0.91	0.82	0.77	0.96	0.36/0.02/0.62	0.375 (XGB)	0.417	0.042
5	0.69	0.46	0.91	0.76	0.74	0.81	0.47/0.25/0.28	0.273 (RF/PINN)	0.412	0.139

However, the improvement in *R*^2^ over the best single model depends not only on whether a complementary model is engaged, but also on how much of the dominant model's error is amenable to cancellation. When the ensemble degenerates to a single model, no cancellation is possible, and the gain is negligible, as shown in Fold 3. With two active models, a single pair of decorrelated error streams yields a modest improvement (Fold 1 and Fold 2), which can be somewhat larger when the dominant model itself leaves considerable room for improvement (Fold 4). The most substantial gain arises only when all three learners receive meaningful weights, enabling multiple pairwise cancellation channels simultaneously (Fold 5). The ensemble thus functions by adaptively canceling the portion of prediction error that arises from domain shift and is unevenly expressed across different architectures. Its value is greatest where the single best model struggles and multiple models provide diverse, mutually decorrelated errors.

These distinct modes of ensemble behavior are inextricably linked to the substantial inter-fold variability discussed in Section 3.3: performance fluctuates dramatically because training literature sources containing unique experimental signatures may not be well represented by others in test sources. The ensemble compensates for this variability by adaptively rebalancing model contributions: it simplifies to a single-model configuration when the domain shift is severe, and expands to a multi-model configuration by exploiting multiple decorrelated error streams when the training data maintain at least partial representativeness.

### 4.4 Comparison with other ML models

Before comparing with external benchmarks, it is instructive to examine how the hybrid ensemble was benchmarked against a basic linear regression using only SEI as the predictor. As shown in Table 8, the linear model fails decisively, returning a negative five-fold average *R*^2^ and underperforming the hybrid ensemble in every single fold. This outcome demonstrates that SEI alone, despite its widespread use as a cross-study comparison metric, cannot capture the nonlinear relationships between operating conditions and reaction performance when data are aggregated from multiple independent sources. In contrast, the hybrid ensemble delivers positive *R*^2^ values in five folds and a near-zero value in the most challenging split. This internal comparison demonstrates that the hybrid ensemble achieves both substantially higher accuracy and markedly better stability. The moderate increase in model complexity is therefore a necessary investment to attain meaningful predictive performance on this multi-source dataset.

**Table 8:** Comparison of the hybrid model and linear regression model

*R* ^2^	Hybrid model	Linear regression
Fold 1	0.562	−0.851
Fold 2	0.791	0.289
Fold 3	0.024	−0.887
Fold 4	0.417	−0.138
Fold 5	0.412	−4.231
Group 5-fold average	0.441	−1.164

Table 9 presents the hybrid ensemble within the landscape of recent ML modeling efforts on plasma-catalytic and related systems. Cai *et al.* applied a hybrid combination of ANN, RT, and SVR to a homogeneous dataset of approximately 100 in-house experiments on plasma-catalytic DRM, reporting an *R*^2^ above 0.98 under a 10-fold CV framework and an *R*^2^ of 0.92 on five new data points.^42^ However, the training data originated from a single experimental setup, which likely limits the model's transferability to different reactor configurations. Wang *et al.* developed a single-algorithm ANN model for catalytic tar reforming using 584 literature data points, achieving an *R*^2^ of 0.96 under a 5-fold CV framework but a markedly lower *R*^2^ of 0.72 on 193 unseen data, a drop the authors attributed to catalyst diversity insufficiently represented in the training set.^81^ Lan *et al.* evaluated eight regressors on 224 literature data points from 21 discharge configurations for nitrogen fixation;^82^ the best stacking ensemble, led by XGB as a meta-learner, achieved a test *R*^2^ of 0.966 but was validated on only two new cases (*R*^2^ = 0.98).

**Table 9:** Comparison of the developed ML method with existing ML methods

Dataset	System	ML models	Performance	Generalizability	Ref.
358 Lit_data	Plasma-based CO_2_ splitting (DBD)	ANN + RF + XGB	*R* ^2^ = 0.791 (best-fold); 0.441 (Group 5-fold CV)	*R* ^2^ = 0.92 (10 new data)	This work
100 Exp_data	Plasma-catalytic DRM (DBD)	ANN + RT + SVR	*R* ^2^ > 0.98 (10-fold CV)	*R* ^2^ = 0.92 (5 new data)	42
584 Lit_data	Catalytic tar reforming	Only ANN model	*R* ^2^ = 0.96 (5-fold CV)	*R* ^2^ = 0.72 (193 new data)	81
224 Lit_data	Plasma-based nitrogen fixation (multi-reactors)	XGB (best model)	*R* ^2^ = 0.966 (5-fold CV)	*R* ^2^ = 0.98 (2 new data)	82

Compared with prior efforts, the present work is distinguished by several methodological refinements that strengthen the reliability and interpretability of the modeling. The model is developed on a larger and explicitly multi-source compilation (358 data points) that covers a substantially wider range of reactor configurations and operating conditions, with a rigorous source-holdout CV framework that treats entire experimental studies as unseen domains, offering a structurally honest estimate of generalization. It further integrates thermodynamic constraints *via* a PINN to prevent unphysical predictions in sparsely sampled regimes; an important safeguard incorporated in ML models applied to plasma-based CO_2_ conversion. The resulting ensemble, while achieving competitive predictive accuracy, additionally provides a more structurally honest estimate of cross-laboratory transferability and an explicit analysis of the conditions under which performance gains from complementary base learners are realized or bounded by irreducible inter-source heterogeneity.

### 4.5 Critical view of the ML model applied in plasma-based gas conversion

Despite growing interest in applying ML to plasma-based gas conversion, the practical benefit of these models for guiding process optimization beyond reported datasets remains limited. A critical examination reveals that the most fundamental bottleneck is not algorithmic but infrastructural.^83^ The plasma-chemical community currently lacks standardized, machine-readable databases that include the physical descriptors essential for capturing discharge mechanisms (*e.g.*, mean electron energy, electron density) and, when relevant, catalyst properties (*e.g.*, surface area and metal dispersion).^12^ Without such information, models are forced to rely on indirect operational parameters whose variations often reflect experimental design choices rather than independent physical drivers. The adoption of FAIR (Findable, Accessible, Interoperable, and Reusable) data principles is therefore an essential first step toward building the comprehensive, richly characterized datasets that robust and transferable models require.^84^ Until such infrastructure exists, even the most sophisticated algorithms face a hard ceiling imposed by the information content of their inputs.

Within this data-limited regime, most current purely data-driven ML models function, by construction, as high-fidelity interpolators: they learn complex functions that faithfully reproduce known experimental trends within the parameter range covered by their training data. While this capability is valuable for screening and optimization within familiar territory, it does not constitute prediction in a mechanistic sense, and offers limited capacity for extrapolation or for uncovering physical mechanisms not already implicit in the data. In such cases, the primary role of the ML model is to find an empirical mapping that accurately reproduces known results, rather than to provide insights into the underlying chemical and physical processes. The resulting models are thus best regarded as powerful interpolative tools, not as predictive frameworks that generate knowledge beyond the information content of their training data.

Precisely because its predictive reliability is confined to the explored domain, the model can produce physically inadmissible outputs when extrapolating to underrepresented regimes. Our initial effort to incorporate physical knowledge through PINN directly addresses this limitation by embedding thermodynamic constraints and thus provides a feasibility guarantee that purely data-driven models lack. While this does not yet yield accurate pointwise predictions on EE in the extrapolation regime, it demonstrates that even simple physical guardrails can extend the trustworthiness of model outputs beyond the strict interpolation envelope. More ambitious integrations of physics and data are emerging in adjacent fields, such as the neural master equation framework for plasma-surface interactions, which retains the structure of governing kinetic equations while using neural networks to represent unknown state transitions.^85^ Such approaches suggest a path forward in the transition of ML from a powerful interpolative tool into a genuine partner in scientific discovery for plasma-based gas conversion.^86^

## 5. Conclusions

We developed a robust and interpretable ML framework for predicting CO_2_ conversion and EE in DBD reactors, trained on a comprehensive database of 357 literature observations spanning 2010 to 2025. A hybrid ensemble model integrating PINN, RF, and XGB algorithms consistently outperformed the best individual model under a source-holdout group 5-fold cross-validation framework, achieving a 21.8% relative improvement in average *R*^2^. Error correlation analysis established that ensemble weights are determined by the pairwise error correlation structure of the base models and their individual predictive accuracy, while the magnitude of improvement over individual models is governed by the degree of error decorrelation and the amount of cancellable error. The ensemble model further achieved an *R*^2^ of 0.92 on unseen data within the explored domain, while the physics-informed loss eliminated unphysical predictions in data-sparse regimes, yielding strictly non-negative CO_2_ conversion estimates. SHAP analysis identified flow rate and power as the dominant features, collectively accounting for 61%–71% of the model's predictions, subject to the statistical associations conditioned on the training data distribution. Overall, this work provides a transparent, rigorously validated baseline and highlights that further progress toward genuinely predictive ML in plasma-based gas conversion processes will benefit from community-wide adoption of FAIR data standards and the development of physics-informed architectures that embed mechanistic constraints directly into the advanced learning framework.

## Author contributions

Conceptualization, J. L. and S. L.; methodology & investigation, J. L., J. X. and P. A.; writing – original draft, J. L. and S. L.; writing – review & editing, J. L., X. L. and A. B.; funding acquisition, F. G., S. L. and A. B.; and supervision, A. B.

## Conflicts of interest

The authors declare that they have no known competing financial interests or personal relationships that could have appeared to influence the work reported in this paper.

## Supplementary Material

GC-028-D6GC01077F-s001

## Data Availability

All data that support the findings of this study are included within the article and its supplementary information (SI). Supplementary information is available. See DOI: https://doi.org/10.1039/d6gc01077f.
