# Supplementary material for: Machine learning to predict plasma-based CO2 conversion in dielectric barrier discharge reactors
Source: Green Chem. 2026 May 14;28(23):9804–21. doi: 10.1039/d6gc01077f (PMC13216855; doi:10.1039/d6gc01077f)
Supplement: GC-028-D6GC01077F-s001 [file GC-028-D6GC01077F-s001.pdf]

Supplementary Information (SI) for Green Chemistry.
This journal is © The Royal Society of Chemistry 2026

Supplementary Information

Machine learning to predict plasma-based CO_2 conversion in dielectric barrier discharges reactors

Jiayin Li[1, 2], Xinpei Lu[3], Pranav Arun[4], Jing Xu[5], Fausto Gallucci[4], Sirui Li[4,*], and Annemie Bogaerts[1, 2,*]

[1] Research Group PLASMANT and Center of Excellence PLASMA, University of Antwerp, Department of Chemistry, Antwerp, 2610, Belgium

[2] Electrification Institute, University of Antwerp, Olieweg 97, 2020 Antwerp, Belgium.

[3] School of Electrical and Electronic Engineering, Huazhong University of Science and Technology, Wuhan, Hubei 430074, China

[4] Department of Chemical Engineering and Chemistry, Eindhoven University of Technology, Eindhoven 5612 AZ, the Netherlands

[5] School of Electronic Information and Communications, Huazhong University of Science and Technology, Wuhan, Hubei, 430074, People's Republic of China

Corresponding author: S.Li1@tue.nl (S. Li), annemie.bogaerts@uantwerpen.be (A. Bogaerts)

Contents

S1. List of all abbreviations in the paper..3

S2. Database for the ML model development..4

S3. Hyperparameters optimization - Bayesian Optimization...................12

S4. Training curves of supervised learning models 13

S5. Experimental setup...15

S6. References 16

S1. List of all abbreviations in the paper

Table S1. List of all abbreviations used in the paper

Full name	Abbreviation
Carbon dioxide	CO_2
Non-thermal plasma	NTP
Power-to-X	P2X
Dielectric barrier discharge	DBD
Energy efficiency	EE
Machine learning	ML
Supervised learning	SL
Unsupervised learning	UL
Reinforcement learning	RL
Active learning	AL
Artificial neural network	ANN
Dry reforming of methane	DRM
Backpropagation	BP
Coefficient of determination	R^2
Bayesian optimization	BO
Specific energy input	SEI
Explainable artificial intelligence	XAI
SHapley Additive exPlanations	SHAP
Pearson's correlation coefficient	PCC
Physics-informed neural network	PINN
Random Forest	RF
Xtreme Gradient Boost	XGB
Mean square error	MSE
Cross-Validation	CV
Mean-absolute error	MAE
Root mean square error	RMSE
Findable, Accessible, Interoperable, and Reusable	FAIR

S2. Database for the ML model development

Table S2. Literature datasets for the plasma-based CO_2 splitting process

No.	Power (W)	Flow rate (mL/min)	Discharge gap (mm)	Reactor length (mm)	Frequency (kHz)	Dielectric constant[1]	CO_2 conversion (%)	Energy efficiency (%)	Ref.
1	100	50	2	9	10	4	24.43	2.38	Ref.[2]
2	150	50	2	9	10	4	24.91	1.618	
3	100	50	2	9	30	4	21.9	2.13	
4	150	50	2	9	30	4	25.81	1.67	
5	200	50	2	9	30	4	27.25	1.32	
6	100	50	2	9	60	4	21.12	2.05	
7	150	50	2	9	60	4	25.58	1.66	
8	200	50	2	9	60	4	30.04	1.46	
9	150	50	2	9	90	4	23.38	1.52	
10	200	50	2	9	90	4	24.35	1.18	
11	21.6	40	2	7.5	13	10	6.8	2.45	Ref.[3]
12	21.6	40	2	11.2	13	10	6.8	2.45	
13	21.6	40	2	15	13	10	6.8	2.45	
14	23.8	40	2	7.5	13	10	7.5	2.45	
15	23.8	40	2	11.2	13	10	8.1	2.65	
16	23.8	40	2	15	13	10	9	2.94	
17	26.5	40	2	7.5	13	10	9.3	2.73	
18	26.5	40	2	11.2	13	10	9.9	2.91	
19	26.5	40	2	15	13	10	10.2	2.99	
20	30.3	40	2	7.5	13	10	11.4	2.93	
21	30.3	40	2	11.2	13	10	11.7	3.00	
22	30.3	40	2	15	13	10	11.5	2.95	
23	35.3	40	2	7.5	13	10	12.6	2.78	
24	35.3	40	2	11.2	13	10	12.6	2.78	
25	35.3	40	2	15	13	10	12.5	2.75	
26	150	300	1	12	120	4	3.5	1.36	Ref.[4]
27	400	300	1	12	120	4	6.3	0.92	
28	600	300	1	12	120	4	8	0.78	
29	1000	300	1	12	120	4	10.5	0.61	
30	15.8	41.9	2.5	15	0.05	4	14.3	7.38	Ref.[5]
31	16.2	41.4	2.5	15	0.05	4	14.5	7.21	
32	15.2	39.5	2.5	15	0.05	4	14.4	7.29	
33	14.7	41.8	2.5	15	0.05	4	13.8	7.63	
34	15.3	38.2	2.5	15	0.05	4	14.7	7.14	

35	9.5	200	2	10	28.6	10	3.2	13.10	Ref.[6]
36	21	200	2	10	28.6	10	7.5	13.89	
37	32	200	2	10	28.6	10	11	13.37	
38	41	200	2	10	28.6	10	14	13.28	
39	54	200	2	10	28.6	10	17.5	12.61	
40	65	200	2	10	28.6	10	20	11.97	
41	77	200	2	10	28.6	10	23	11.62	
42	87	200	2	10	28.6	10	25.2	11.27	
43	95	200	2	10	28.6	10	28	11.46	
44	10	25	2.5	10	9	4	17.4	8.46	Ref.[7]
45	20	25	2.5	10	9	4	19.8	4.81	
46	30	25	2.5	10	9	4	21	3.40	
47	40	25	2.5	10	9	4	22	2.67	
48	50	25	2.5	10	9	4	22.4	2.18	
49	50	31.2	2.5	10	9	4	20.8	2.52	
50	50	41.2	2.5	10	9	4	18	2.88	
51	50	62.5	2.5	10	9	4	15.8	3.84	
52	50	125	2.5	10	9	4	12.6	6.13	
53	30	50	2	19	9	4	2	0.65	Ref.[8]
54	40	50	2	19	9	4	3	0.73	
55	50	50	2	19	9	4	5	0.97	
56	60	50	2	19	9	4	6	0.97	
57	27.35	150	1	1	8.1	4	3.5	3.73	Ref.[9]
58	27.35	150	1	2.5	8.1	4	8.8	9.39	
59	27.35	150	1	4	8.1	4	10.7	11.41	
60	27.35	150	1	5.5	8.1	4	14	14.93	
61	27.35	150	1	7	8.1	4	15	16.00	
62	27.35	30	1	7	8.1	4	18.6	3.97	
63	27.35	50	1	7	8.1	4	18.4	6.54	
64	27.35	100	1	7	8.1	4	17.8	12.66	
65	27.35	200	1	7	8.1	4	10.8	15.36	
66	27.35	250	1	7	8.1	4	8.4	14.93	
67	27.35	300	1	7	8.1	4	6.7	14.29	
68	20	50	3	5.7	9	4	7.4	3.60	Ref.[10]
69	30	50	3	5.7	9	4	10.6	3.44	
70	40	50	3	5.7	9	4	13	3.16	
71	50	50	3	5.7	9	4	16	3.11	
72	6.38	43.7	0.5	6	9	4	2.04884	2.73	Ref.[11]
73	12.66	43.7	0.5	6	9	4	4.79561	3.22	
74	20.93	43.7	0.5	6	9	4	6.54442	2.66	
75	25.82	43.7	0.5	6	9	4	7.63426	2.51	
76	33.93	43.7	0.5	6	9	4	9.36723	2.35	

77	50	50	2	10	28.6	9.6	35	6.74	Ref.[12]
78	50	100	2	10	28.6	9.6	31	11.9	
79	50	200	2	10	28.6	9.6	26	20.0	
80	50	300	2	10	28.6	9.6	16	18.5	
81	50	400	2	10	28.6	9.6	11	16.9	
82	50	500	2	10	28.6	9.6	7	13.5	
83	50	1000	2	10	28.6	9.6	4	15.4	
84	50	2000	2	10	28.6	9.6	2	15.4	
85	50	3000	2	10	28.6	4.6	0.87	11.6	
86	9.5	200	2	10	28.6	4.6	1.74	7.09	
87	20	200	2	10	28.6	4.6	3.5	6.86	
88	32	200	2	10	28.6	4.6	5.98	7.19	
89	42	200	2	10	28.6	4.6	7.89	7.15	
90	52.1	200	2	10	28.6	4.6	8.6	6.31	
91	61.1	200	2	10	28.6	4.6	10.13	6.33	
92	75	200	2	10	28.6	4.6	11.75	6.01	
93	74	200	2	10	27.1	9.6	24.34	12.58	
94	74	200	2	10	27.1	6	21.39	11.04	
95	74	200	2	10	27.1	4.6	20.7	10.72	
96	74	200	2	10	27.1	3.8	24.29	12.56	
97	10	25	3	10	9	4	16.2	7.88	Ref.[13]
98	20	25	3	10	9	4	18.2	4.42	
99	30	25	3	10	9	4	19.6	3.18	
100	40	25	3	10	9	4	20.4	2.48	
101	50	25	3	10	9	4	20.8	2.02	
102	10	25	3.5	10	9	4	13.8	6.71	
103	20	25	3.5	10	9	4	14.9	3.62	
104	30	25	3.5	10	9	4	15.8	2.56	
105	40	25	3.5	10	9	4	16.6	2.02	
106	50	25	3.5	10	9	4	17.4	1.69	
107	10	25	2.5	6	9	4	15.8	7.68	
108	20	25	2.5	6	9	4	17.2	4.18	
109	30	25	2.5	6	9	4	18.2	2.95	
110	40	25	2.5	6	9	4	18.8	2.29	
111	50	25	2.5	6	9	4	19.6	1.91	
112	10	25	2.5	14	9	4	18.8	9.14	
113	20	25	2.5	14	9	4	20.8	5.06	
114	30	25	2.5	14	9	4	22.4	3.63	
115	40	25	2.5	14	9	4	24.1	2.93	
116	50	25	2.5	14	9	4	24.8	2.41	
117	40	25	2.5	10	8	4	22.85	2.98	
118	40	25	2.5	10	10	4	22.37	2.91	
119	40	25	2.5	10	11	4	21.92	2.86	

120	35	500	1.8	9	23.5	4	2.65	7.76	Ref.[14]
121	35	500	2.3	9	23.5	4	2.07	6.12	
122	35	500	3.3	9	23.5	4	2.05	5.94	
123	35	105	1.8	9	23.5	4	9.08	5.55	
124	35	105	2.3	9	23.5	4	11.64	7.5	
125	35	88	3.3	9	23.5	4	7.6	4.20	
126	35	50	1.8	9	23.5	4	16.37	5	
127	35	50	2.3	9	23.5	4	17.97	5.55	
128	35	43	3.3	9	23.5	4	10.9	2.88	
129	35	25	1.8	9	23.5	4	28.36	4.16	
130	35	25	2.3	9	23.5	4	25.36	3.87	
131	35	23	3.3	9	23.5	4	15.79	2.15	
132	35	20	1.8	9	23.5	4	30.46	3.795	
133	35	20	2.3	9	23.5	4	29.26	3.6	
134	35	18	3.3	9	23.5	4	17.08	1.86	
135	35	15	1.8	9	23.5	4	31.76	2.84	
136	35	15	2.3	9	23.5	4	31.66	3.03	
137	35	15	3.3	9	23.5	4	19.98	1.74	
138	35	10	1.8	9	23.5	4	33.06	1.97	
139	35	10	2.3	9	23.5	4	32.25	1.97	
140	35	10	3.3	9	23.5	4	23.26	1.1	
141	40	444	1.8	9	23.5	9.8	2.89	7.99	
142	40	111	1.8	9	23.5	9.8	13.92	7.36	
143	40	49	1.8	9	23.5	9.8	19.94	5.43	
144	40	32	1.8	9	23.5	9.8	24.86	4.31	
145	40	25	1.8	9	23.5	9.8	28.66	3.95	
146	40	20	1.8	9	23.5	9.8	30.93	3.44	
147	40	15	1.8	9	23.5	9.8	32.96	2.74	
148	40	10	1.8	9	23.5	9.8	34.02	1.83	
149	15	50	1.8	9	23.5	9.8	13.1	7.87	
150	38	50	1.8	9	23.5	9.8	20.05	5.42	
151	60	50	1.8	9	23.5	9.8	24.12	4.25	
152	72	50	1.8	9	23.5	9.8	24.92	3.36	
153	96	50	1.8	9	23.5	9.8	25.19	2.61	
154	114	50	1.8	9	23.5	9.8	24.92	2.24	
155	133	50	1.8	9	23.5	9.8	25.08	2.22	
156	17	100	1.8	9	23.5	9.8	8.56	9.27	
157	40	100	1.8	9	23.5	9.8	13.86	7.38	
158	58	100	1.8	9	23.5	9.8	16.82	5.76	
159	80	100	1.8	9	23.5	9.8	18.49	4.98	
160	97	100	1.8	9	23.5	9.8	18.87	4.09	
161	113	100	1.8	9	23.5	9.8	19.64	3.56	
162	128	100	1.8	9	23.5	9.8	19.56	3.12	

163	20	25	8	10	12	4.6	17.46	2.9	Ref.[15]
164	20	35	8	10	12	4.6	18.58	2.21	
165	20	45	8	10	12	4.6	20.28	1.89	
166	20	55	8	10	12	4.6	24.5	1.8	
167	20	65	8	10	12	4.6	24.85	1.58	
168	20	25	8	15	12	4.6	17.54	2.91	
169	20	35	8	15	12	4.6	19.11	2.28	
170	20	45	8	15	12	4.6	22.55	2.08	
171	20	55	8	15	12	4.6	25.14	1.89	
172	20	65	8	15	12	4.6	25.51	1.63	
173	20	25	8	20	12	4.6	18.65	3.12	
174	20	35	8	20	12	4.6	20.65	2.46	
175	20	45	8	20	12	4.6	24.67	2.28	
176	20	55	8	20	12	4.6	26.15	1.97	
177	20	65	8	20	12	4.6	26.64	1.71	
178	20	30	5	21	14	4	12.78	1.65	Ref.[16]
179	20	50	5	21	14	4	15.61	1.22	
180	20	70	5	21	14	4	16.6	0.92	
181	20	90	5	21	14	4	16.03	0.68	
182	20	110	5	21	14	4	15.83	0.55	
183	40	150	2.5	25	8.8	4	3.77	5.33	Ref.[17]
184	60	150	2.5	25	8.8	4	4.09	3.33	
185	80	150	2.5	25	8.8	4	4.25	2.65	
186	100	150	2.5	25	8.8	4	5	2.5	
187	40	300	2.5	25	8.8	4	2.33	6.31	
188	40	450	2.5	25	8.8	4	1.68	7.1	
189	40	600	2.5	25	8.8	4	1.06	7.63	
190	34.38	100	0.25	40	10	3.7	10.69	6.46	Ref.[18]
191	55.73	100	0.25	40	10	3.7	15.17	5.67	
192	78.91	100	0.25	40	10	3.7	18.15	4.79	
193	101.3	100	0.25	40	10	3.7	20.24	4.17	
194	50	10	0.25	40	10	3.7	45.36	1.79	
195	50	20	0.25	40	10	3.7	51.45	4.15	
196	50	50	0.25	40	10	3.7	32.69	7.25	
197	50	100	0.25	40	10	3.7	15.14	5.67	
198	27	20	0.25	40	10	3.7	43.46	6.64	
199	51.5	20	0.25	40	10	3.7	51.47	4.16	
200	73.6	20	0.25	40	10	3.7	50.97	2.89	
201	90.7	20	0.25	40	10	3.7	48.11	2.14	
202	20.8	50	0.25	40	10	3.7	19.15	9.44	
203	46.8	50	0.25	40	10	3.7	32.58	7.25	
204	73.8	50	0.25	40	10	3.7	37.54	5.29	
205	94.5	50	0.25	40	10	3.7	38.68	4.25	

206	30	20	1.5	8	7.1	4	10.09	1.41	Ref.[19]
207	30	40	1.5	8	7.1	4	7.08	1.99	
208	30	60	1.5	8	7.1	4	5.51	2.32	
209	30	80	1.5	8	7.1	4	4.53	2.55	
210	30	100	1.5	8	7.1	4	4	2.8	
211	100	50	4.5	10	23.5	10	11	1.87	Ref.[20]
212	100	192	4.5	10	23.5	10	5.27	3.22	
213	100	50	1.23	10	23.5	10	30.17	4.69	
214	100	50	0.705	10	23.5	10	35.5	5.52	
215	100	50	0.455	10	23.5	10	50.59	7.87	
216	100	50	0.268	10	23.5	10	53.58	8.33	
217	0.5	30	6	12	0.05	4	1.21	14.12	Ref.[21]
218	1	30	6	12	0.05	4	2.91	16.97	
219	1.4	30	6	12	0.05	4	5.3	22.09	
220	1.8	30	6	12	0.05	4	6.24	20.23	
221	2.2	30	6	12	0.05	4	7.44	19.73	
222	0.63	30	4.5	11	0.05	4	2.4	22.23	Ref.[22]
223	0.89	30	4.5	11	0.05	4	3.06	20.06	
224	1.1	30	4.5	11	0.05	4	4.25	22.54	
225	1.3	30	4.5	11	0.05	4	5.2	23.34	
226	10	20	0.6	8	18	4	9.27	3.59	Ref.[23]
227	15	20	0.6	8	18	4	14.54	3.58	
228	20	20	0.6	8	18	4	14.32	2.7	
229	25	20	0.6	8	18	4	14.38	2.18	
230	14.5	10	4	8	8.8	4	13.08	1.91	Ref.[24]
231	14.5	20	4	8	8.8	4	10.88	3.11	
232	14.5	30	4	8	8.8	4	8.95	3.8	
233	14.5	40	4	8	8.8	4	8.72	5	
234	14.5	50	4	8	8.8	4	7.45	5.39	
235	8	60	2.5	15	0.05	4	6.7	9.77	Ref.[25]
236	8	45	2.5	15	0.05	4	8.22	8.99	
237	8	35	2.5	15	0.05	4	9.58	8.15	
238	8	30	2.5	15	0.05	4	10.51	7.67	
239	8	25	2.5	15	0.05	4	12.96	7.88	
240	8	24	2.5	15	0.05	4	10.87	6.34	
241	8	17	2.5	15	0.05	4	15.22	6.29	
242	8	15	2.5	15	0.05	4	13.93	5.08	
243	10	15	2.5	15	0.05	4	17.27	5.04	
244	14	15	2.5	15	0.05	4	17.29	3.60	
245	20	15	2.5	15	0.05	4	21.72	3.17	

246	10	20	1	20	40	4	11.89	4.63	Ref.[26]
247	20	20	1	20	40	4	17.23	3.35	
248	30	20	1	20	40	4	19.85	2.57	
249	40	20	1	20	40	4	21.62	2.10	
250	50	20	1	20	40	4	22.05	1.72	
251	30	11.52	0.268	10	3	10	33.34	2.68	Ref.[27]
252	30	19.35	0.455	10	3	10	30	3.67	
253	30	29.53	0.705	10	3	10	18	3.55	
254	30	50	1.23	10	3	10	12.8	4.38	
255	30	150.18	4.705	10	3	10	4.3	4.46	
256	30	50	0.268	10	3	10	12.51	4.38	
257	30	50	0.455	10	3	10	13.43	4.7	
258	30	50	0.705	10	3	10	13.47	4.71	
259	30	50	4.705	10	3	10	8.06	2.82	
260	30	2	0.455	10	3	10	53.53	0.75	
261	30	2.6	0.455	10	3	10	54.32	0.94	
262	30	3	0.455	10	3	10	54.49	1.15	
263	30	3.57	0.455	10	3	10	53.12	1.28	
264	30	5	0.455	10	3	10	50.66	1.83	
265	30	7.5	0.455	10	3	10	47.74	2.5	
266	30	10	0.455	10	3	10	39.32	2.87	
267	30	30	0.455	10	3	10	21.61	4.61	
268	30	40	0.455	10	3	10	16.4	4.68	
269	22.30	100	1.05	10	45	9.6	6.09	5.31	Ref.[28]
270	22.68	80	1.05	10	45	9.6	7.58	5.20	
271	22.14	60	1.05	10	45	9.6	9.59	5.05	
272	21.70	40	1.05	10	45	9.6	13.27	4.76	
273	12.06	20	1.05	10	45	9.6	13.96	4.50	
274	17.72	20	1.05	10	45	9.6	19.18	4.21	
275	21.97	20	1.05	10	45	9.6	22.03	3.90	
276	26.64	20	1.05	10	45	9.6	23.07	3.37	
277	30.66	20	1.05	10	45	9.6	24.70	3.13	
278	22.98	100	1.05	10	45	9.6	6.29	5.33	
279	22.76	80	1.05	10	45	9.6	7.67	5.24	
280	22.41	60	1.05	10	45	9.6	9.60	5.00	
281	22.43	40	1.05	10	45	9.6	13.37	4.64	
282	12.92	20	1.05	10	45	9.6	14.84	4.47	
283	17.78	20	1.05	10	45	9.6	19.59	4.29	
284	21.99	20	1.05	10	45	9.6	21.90	3.87	
285	25.89	20	1.05	10	45	9.6	24.16	3.63	
286	29.87	20	1.05	10	45	9.6	26.05	3.39	
287	18.88	100	0.8	10	45	9.6	5.54	5.71	
288	18.37	80	0.8	10	45	9.6	7.11	6.02	
289	17.71	60	0.8	10	45	9.6	8.60	5.66	Ref.[28]

290	6.12	20	0.8	10	45	9.6	2.22	1.41	
291	17.10	40	0.8	10	45	9.6	11.93	5.43	
292	14.10	20	0.8	10	45	9.6	13.72	3.79	
293	17.97	20	0.8	10	45	9.6	18.67	4.04	
294	22.99	20	0.8	10	45	9.6	25.36	4.29	
295	26.15	20	0.8	10	45	9.6	25.37	3.77	
296	18.14	100	0.8	10	45	9.6	5.48	5.88	
297	17.67	80	0.8	10	45	9.6	6.62	5.83	
298	16.75	60	0.8	10	45	9.6	8.00	5.57	
299	8.03	20	0.8	10	45	9.6	10.51	5.09	
300	16.77	40	0.8	10	45	9.6	11.86	5.50	
301	15.20	20	0.8	10	45	9.6	16.21	4.15	
302	17.91	20	0.8	10	45	9.6	20.94	4.55	
303	20.86	20	0.8	10	45	9.6	22.84	4.26	
304	24.70	20	0.8	10	45	9.6	25.79	4.06	
305	24.86	100	1.3	10	45	9.6	6.47	5.06	
306	24.74	80	1.3	10	45	9.6	7.85	4.94	
307	23.58	60	1.3	10	45	9.6	9.84	4.87	
308	23.68	40	1.3	10	45	9.6	13.72	4.51	
309	13.76	20	1.3	10	45	9.6	14.02	3.96	
310	18.81	20	1.3	10	45	9.6	19.32	4.00	
311	23.44	20	1.3	10	45	9.6	21.42	3.55	
312	27.12	20	1.3	10	45	9.6	23.58	3.38	
313	31.28	20	1.3	10	45	9.6	25.59	3.18	
314	22.91	100	1.3	10	45	9.6	6.15	5.22	
315	22.77	80	1.3	10	45	9.6	7.59	5.18	
316	22.85	60	1.3	10	45	9.6	9.60	4.90	
317	22.65	40	1.3	10	45	9.6	13.40	4.60	
318	13.32	20	1.3	10	45	9.6	14.78	4.31	
319	18.04	20	1.3	10	45	9.6	18.96	4.09	
320	22.60	20	1.3	10	45	9.6	21.71	3.74	
321	26.22	20	1.3	10	45	9.6	23.37	3.47	
322	30.81	20	1.3	10	45	9.6	24.86	3.14	
323	27.94	100	1.3	7.5	45	9.6	7.14	4.97	
324	27.28	80	1.3	7.5	45	9.6	8.46	4.83	
325	26.71	60	1.3	7.5	45	9.6	10.72	4.68	
326	26.50	40	1.3	7.5	45	9.6	14.65	4.30	
327	18.13	20	1.3	7.5	45	9.6	12.77	2.74	
328	21.76	20	1.3	7.5	45	9.6	21.03	3.76	
329	26.62	20	1.3	7.5	45	9.6	23.16	3.38	
330	30.97	20	1.3	7.5	45	9.6	24.77	3.11	
331	34.53	20	1.3	7.5	45	9.6	24.88	2.80	
332	25.86	100	1.3	7.5	45	9.6	6.76	5.08	
333	25.60	80	1.3	7.5	45	9.6	8.25	5.01	

334	25.14	60	1.3	7.5	45	9.6	10.49	4.87	
335	25.38	40	1.3	7.5	45	9.6	14.72	4.51	
336	16.26	20	1.3	7.5	45	9.6	11.16	2.67	
337	20.59	20	1.3	7.5	45	9.6	19.14	3.62	
338	25.59	20	1.3	7.5	45	9.6	22.97	3.49	
339	29.72	20	1.3	7.5	45	9.6	24.03	3.14	
340	34.40	20	1.3	7.5	45	9.6	25.22	2.85	
341	30.18	100	1.3	5	45	9.6	7.29	4.70	
342	30.39	80	1.3	5	45	9.6	8.83	4.52	
343	29.41	60	1.3	5	45	9.6	11.21	4.45	
344	29.24	40	1.3	5	45	9.6	15.06	4.01	
345	19.19	20	1.3	5	45	9.6	19.27	3.91	
346	24.51	20	1.3	5	45	9.6	21.73	3.45	
347	29.89	20	1.3	5	45	9.6	23.19	3.02	
348	34.40	20	1.3	5	45	9.6	23.83	2.69	
349	38.84	20	1.3	5	45	9.6	21.06	2.11	
350	28.61	100	1.3	5	45	9.6	7.21	4.90	
351	28.21	80	1.3	5	45	9.6	8.59	4.74	
352	27.93	60	1.3	5	45	9.6	10.84	4.53	
353	27.31	40	1.3	5	45	9.6	14.76	4.21	
354	18.68	20	1.3	5	45	9.6	18.38	3.83	
355	22.85	20	1.3	5	45	9.6	21.01	3.58	
356	27.78	20	1.3	5	45	9.6	22.95	3.21	
357	31.83	20	1.3	5	45	9.6	24.09	2.94	
358	37.89	20	1.3	5	45	9.6	25.65	2.63	

Note:
(1) We recalculated some reported results from the literature to ensure uniformity for our ML model development. Specific example is standardization of Specific Energy Input (SEI): several studies showed only the SEI value in the figure, defined as the ratio of power to gas flow rate.[14,25] If the authors provided either the SEI along with the power or the flow rate, we used this information to recalculate the missing parameter to standardize the data.
(2) When the dielectric barrier material was identified as alumina or quartz in each study but its relative permittivity was not explicitly reported, we assigned a value of 10 for alumina and 4 for quartz, based on typical values cited in Ref.[1]

S3. Hyperparameters optimization - Bayesian Optimization

Hyperparameters are predefined configurations that govern a model's learning behavior and overall effectiveness. A Bayesian Optimization (BO) framework was employed to systematically search for optimal hyperparameters across three supervised learning (SL) algorithms: Physics-informed neural network (PINN), Random Forest (RF), and Xtreme Gradient Boost (XGB). The objective was to maximize test performance of models, as measured by the R^2 score.

BO is a sequential, model-based tuning strategy built on two core components: a probabilistic surrogate model and an acquisition function.[29] Gaussian Process

Regression (GPR) served as the surrogate model, using an initial set of hyperparameter samples to estimate both the expected R^2 and the associated uncertainty across the search space. These predictions guide the acquisition function, which strategically balances exploration of under-sampled regions with exploitation of areas known to yield high performance. At each iteration, the algorithm selects the hyperparameter set that maximizes this function, progressively refining its approximations as more samples are evaluated. This process continues until convergence toward near-optimal configurations.[30]

For the PINN, architectural hyperparameters, including the number of hidden layers, neurons per layer, and activation function, were optimized within predefined ranges. The RF model's tuning involved key parameters such as the number of estimators, maximum depth, maximum features, minimum samples split, and minimum samples leaf. For the XGB model, the optimization encompassed learning rate, subsample ratio, colsample_bytree, L1 and L2 regularization terms (reg_alpha and reg_lambda), minimum child weight, and gamma. All hyperparameters not explicitly listed in the optimization process were kept at their default values. The finalized hyperparameters Group 5-fold Cross-Validation (CV) framework and random 5-fold CV framework, along with their respective search ranges and selected values, are summarized in Table S3 and Table S4, respectively.

Table S3. Hyperparameters of SL models and optimized ranges under Group 5-fold CV framework.

SL models	Hyperparameter	Optimization range	Optimized values in each fold				
			1	2	3	4	5
PINN	Hidden layer	[2, 3, 4]	3				
	Neurons per layer	[5, 60]	(12, 5, 5)	(45, 38, 20)	(53, 6, 31)	(33, 42, 36)	(10, 26, 5)
	Activation function	tanh, ReLU, logistic	ReLU				
RF	N_estimators	[100, 1000]	100	543	458	100	100
	Max_depth	[None, 5, 8, 10, 12, 15, 20, 25, 30]	12	8	5	15	5
	Max_features	[0.3, 1]	0.713	0.927	1.000	0.400	1.000
	Min_samples_split	[2, 20]	2	5	2	2	2
	Min_samples_leaf	[1, 10]	4	2	10	1	1
XGB	N_estimators	[100, 1000]	706	1000	1000	1000	1000
	Max_depth	[3, 10]	10	3	10	10	10
	Learning_rate	[0.005, 0.3]	0.005	0.086	0.3	0.21	0.005
	Subsample	[0.6, 1]	0.6	1.0	0.6	0.76	0.6
	Colsample_bytree	[0.6, 1]	1.0	0.6	1.0	0.70	1.0
	Reg_lambda	[0.01, 100]	100	0.959	0.01	0.024	0.01
	Reg_alpha	[0.001 ,10]	0.001				
	Min_child_weight	[1, 20]	2	4	1	4	1
	Gamma	[0, 5]	0.0	0.735	5.0	0.03	5.0

Table S4. Hyperparameters of SL models and optimized ranges under 5-fold CV framework.

SL models	Hyperparameter	Optimization range	Optimized value
PINN	Hidden layer	[2, 3, 4]	3
	Neurons per layer	[2, 100]	(60, 21, 40)
	Activation	tanh, ReLU, logistic	ReLU
RF	N_estimators	[100, 1000]	1000
	Max_depth	[None, 5, 8, 10, 12, 15, 20, 25, 30]	None
	Max_features	[0.3, 1]	0.3
	Min_samples_split	[2, 20]	2
	Min_samples_leaf	[1, 10]	1
XGB	N_estimators	[100, 1000]	1000
	Max_depth	[3, 10]	10
	Learning_rate	[0.005, 0.3]	0.3
	Subsample	[0.6, 1]	0.6
	Colsample_bytree	[0.6, 1]	1
	Reg_lambda	[0.01, 100]	100
	Reg_alpha	[0.001 ,10]	0.001
	Min_child_weight	[1, 20]	1
	Gamma	[0, 5]	0.00

S4. Training curves of supervised learning models

As depicted in Fig. S1, the training process for PINN model and XGB model exhibits a steady decline in mean squared error (MSE) under the Group 5-fold cross-validation (CV) framework. Following loss computation, the backpropagation (BP) algorithm employs the chain rule to compute the partial derivatives of the error with respect to each network weight. These gradients subsequently guide gradient descent in iteratively updating the model parameters toward an optimal solution.

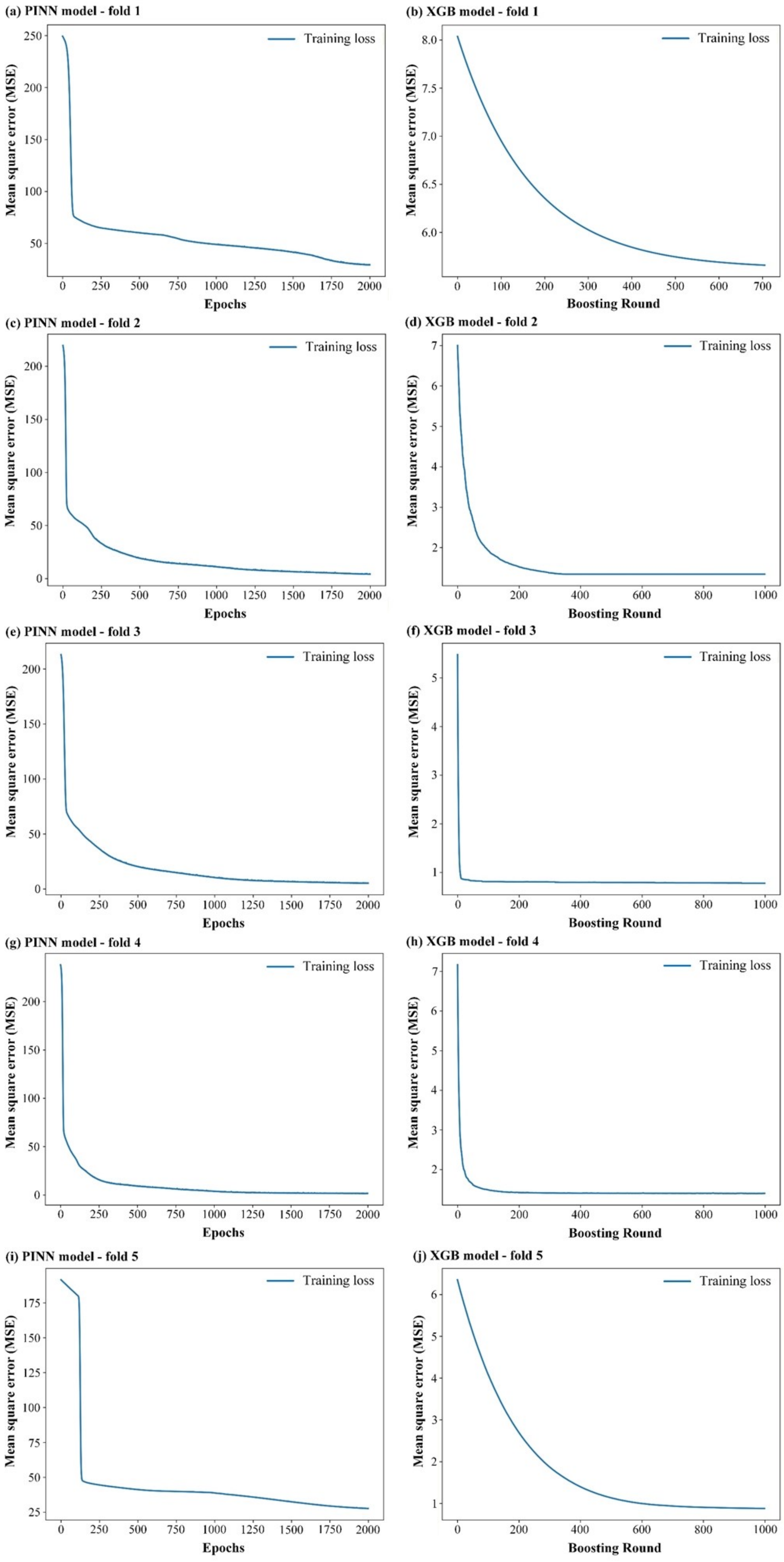

Fig. S1. MSE of the best fitness value in each epoch and boosting round for the PINN model and XGB model within the (a, b) Fold 1, (c, d) Fold 2, (e, f) Fold 3, (g, h) Fold 4, and (i, j) Fold 5.

S5. Experimental setup

The ML model was developed using datasets compiled from the literature, and its generalizability was subsequently validated against experimental data obtained in this study. A schematic overview of the experimental setup is provided in Fig. S3.[31] The dielectric barrier discharge reactor consists of an alumina tube with a fixed outer diameter of 8.6 mm. A stainless-steel sheet (100 mm in length) was wrapped around the tube and grounded through a 100 nF capacitor. The inner electrode was a stainless-steel rod with diameters of 6.5 mm and 7.0 mm, corresponding to discharge gaps of 1.05 mm and 0.8 mm, respectively. This electrode was connected to an AC high-voltage power supply (AFS G155−150K) operating at a frequency ranging from 40 kHz to 50 kHz. Voltage and charge waveforms were captured using a four-channel oscilloscope (PicoScope 3405D, 100 MHz bandwidth, 8-bit resolution, 1 G/s sampling rate). The applied voltage across the reactor was measured with a 1:1000 high-voltage probe (Tektronix P6015A), while the charge transferred during plasma discharge was determined by monitoring the voltage across the 100 nF capacitor using a 1:10 probe (Pico TA 131). Discharge power was calculated from the acquired voltage and charge waveforms using the Lissajous figure method.[13]

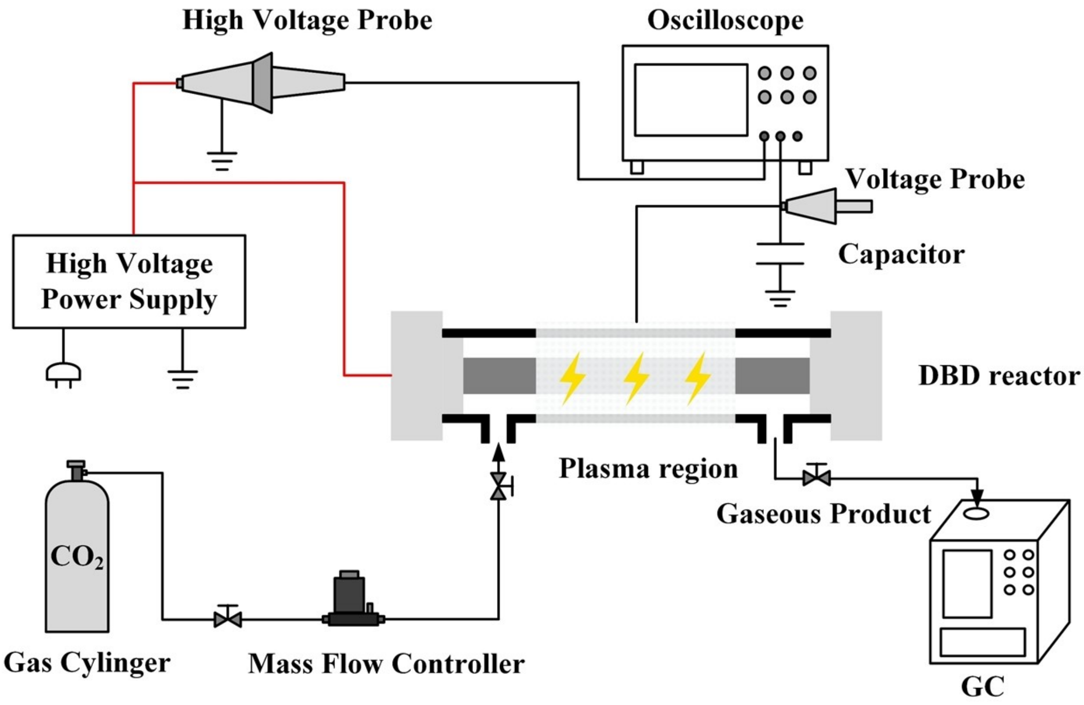

Fig. S2. Schematic overview of the experimental setup.

S6. References

1 David RL, CRC Handbook of chemistry and physics. CRC Press, New York, 1999.

2 S. Paulussen, B. Verheyde, X. Tu, C. De Bie, T. Martens, D. Petrovic, A. Bogaerts and B. Sels, *Plasma Sources Sci. Technol.*, 2010, **19**, 34015.

3 Q. Yu, M. Kong, T. Liu, J. Fei and X. Zheng, *Plasma Chem. Plasma Process.*, 2012, **32**, 153–163.

4 N. Lisi, U. Pasqual Laverdura, R. Chierchia, I. Luisetto and S. Stendardo, *Sci. Rep.*, 2023, **13**, 7394.

5 D. Mei, Y.-L. He, S. Liu, J. Yan and X. Tu, *Plasma Process. Polym.*, 2016, **13**, 544–556.

6 A. Ozkan, T. Dufour, T. Silva, N. Britun, R. Snyders, A. Bogaerts and F. Reniers, *Plasma Sources Sci. Technol.*, 2016, **25**, 25013.

7 M. Alliati, D. Mei and X. Tu, *J. CO2 Util.*, 2018, **27**, 308–319.

8 N. Lu, C. Zhang, K. Shang, N. Jiang, J. Li and Y. Wu, *J. Phys. D: Appl. Phys.*, 2019, **52**, 224003.

9 G. Niu, Y. Qin, W. Li and Y. Duan, *Plasma Chem. Plasma Process.*, 2019, **39**, 809–824.

10 D. Mei, X. Zhu, Y.-L. He, J. D. Yan and X. Tu, *Plasma Sources Sci. Technol.*, 2014, **24**, 15011.

11 B. Wang, X. Wang and B. Zhang, *Front. Chem. Sci. Eng.*, 2021, **15**, 687–697.

12 A. Ozkan, A. Bogaerts and F. Reniers, *J. Phys. D: Appl. Phys.*, 2017, **50**, 84004.

13 D. Mei and X. Tu, *J. CO2 Util.*, 2017, **19**, 68–78.

14 R. Aerts, W. Somers and A. Bogaerts, *ChemSusChem*, 2015, **8**, 702–716.

15 A. Zhou, D. Chen, C. Ma, F. Yu and B. Dai, *Catalysts*, 2018, **8**, 256.

16 J. Li, S. Zhu, K. Lu, C. Ma, D. Yang and F. Yu, *J. Environ. Chem. Eng.*, 2021, **9**, 104654.

17 L. He, X. Yue, X. Liu and Z. Wu, *J. Phys. D: Appl. Phys.*, 2025, **58**, 105204.

18 H. Yukio, P. Emeraldi, T. Imai and S. Kambara, *Int. J. Plasma Environ. Sci. Technol.*, 2023, **17**, e01007.

19 P. Wu, X. Li, N. Ullah and Z. Li, *Mole. Catal.*, 2021, **499**, 111304.

20 I. Michielsen, Y. Uytdenhouwen, J. Pype, B. Michielsen, J. Mertens, F. Reniers, V. Meynen and A. Bogaerts, *Chem. Eng. J.*, 2017, **326**, 477–488.

21 D. Ray, P. Chawdhury, K. V. S. S. Bhargavi, S. Thatikonda, N. Lingaiah and Ch. Subrahmanyam, *J. CO2 Util.*, 2021, **44**, 101400.

22 M. Umamaheswara Rao, K. Bhargavi, G. Madras and Ch. Subrahmanyam, *Chem. Eng. J.*, 2023, **468**, 143671.

23 X. Duan, Z. Hu, Y. Li and B. Wang, *AIChE J.*, 2015, **61**, 898–903.

24 M. Xia, W. Ding, C. Shen, Z. Zhang and C. Liu, *Ind. Eng. Chem. Res.*, 2022, **61**, 10455–10460.

25 D. Mei, X. Zhu, C. Wu, B. Ashford, P. T. Williams and X. Tu, *Appl. Catal. B: Environ.*, 2016, **182**, 525–532.

26 Y. Gao, R. Zhou, B. Chen, L. Xiao, X. Zhao, J. Sun, R. Zhou, J. Zhang and Z. Liu, *ACS Sustainable Chem. Eng.*, 2024, **12**, 10993–11005.

27 Y. Uytdenhouwen, S. Van Alphen, I. Michielsen, V. Meynen, P. Cool and A. Bogaerts, *Chem. Eng. J.*, 2018, **348**, 557–568.

28 J. Li, G. Palma, J. Xu, F. Gallucci, A. Bogaerts and S. Li, *Energy Convers. Manage.* 2026, **356**, 121210.

29 B. Hickish, D. I. Fletcher and R. F. Harrison, *Int. J. Rail Transp*., 2020, **8**, 307–323.

30 S. Greenhill, S. Rana, S. Gupta, P. Vellanki and S. Venkatesh, *IEEE Access*, 2020, **8**, 13937–13948.

31 R. Vertongen, G. De Felice, H. van den Bogaard, F. Gallucci, A. Bogaerts and S. Li, *ACS Sustainable Chem. Eng.*, 2024, **12**, 10841–10853.
